# Stacking-Order-Dependent Excitonic Properties Reveal Interlayer Interactions in Bulk ReS_2_

**DOI:** 10.1021/acsphotonics.3c00477

**Published:** 2023-08-16

**Authors:** Marco van der Laan, Edwin Heemskerk, Floris Kienhuis, Nella Diepeveen, Deepika Poonia, Sachin Kinge, Minh Triet Dang, Van An Dinh, Laurens D. A. Siebbeles, Anna Isaeva, Jorik van de Groep, Peter Schall

**Affiliations:** † Van der Waals-Zeeman Institute, Institute of Physics, University of Amsterdam, Science Park 904, 1098 XH Amsterdam, The Netherlands; ‡ Optoelectronic Materials Section, Department of Chemical Engineering, Delft University of Technology, 2629 HZ Delft, The Netherlands; § Materials Research & Development, Toyota Motor Europe, B1930 Zaventem, Belgium; ∥ School of Education, Can Tho University, 3-2 Road, Can Tho City 900000, Vietnam; ⊥ Department of Precision Engineering, Graduate School of Engineering, Osaka University, 2-1 Yamadaoka, Suita, Osaka 565-0871, Japan; # Leibniz IFW Dresden, Helmholtzstr. 20, D-01069 Dresden, Germany

**Keywords:** optical spectroscopy, 2D materials, excitons, interlayer interactions, anisotropy, binding energy

## Abstract

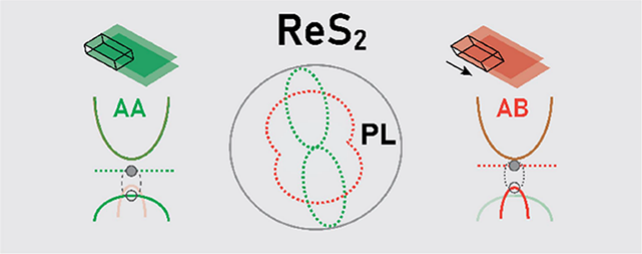

Rhenium disulfide, a member of the transition metal dichalcogenide family of semiconducting materials, is unique among 2D van der Waals materials due to its anisotropy and, albeit weak, interlayer interactions, confining excitons within single atomic layers and leading to monolayer-like excitonic properties even in bulk crystals. While recent work has established the existence of two stacking modes in bulk, AA and AB_,_ the influence of the different interlayer coupling on the excitonic properties has been poorly explored. Here, we use polarization-dependent optical measurements to elucidate the nature of excitons in AA and AB-stacked rhenium disulfide to obtain insight into the effect of interlayer interactions. We combine polarization-dependent Raman with low-temperature photoluminescence and reflection spectroscopy to show that, while the similar polarization dependence of both stacking orders indicates similar excitonic alignments within the crystal planes, differences in peak width, position, and degree of anisotropy reveal a different degree of interlayer coupling. DFT calculations confirm the very similar band structure of the two stacking orders while revealing a change of the spin-split states at the top of the valence band to possibly underlie their different exciton binding energies. These results suggest that the excitonic properties are largely determined by in-plane interactions, however, strongly modified by the interlayer coupling. These modifications are stronger than those in other 2D semiconductors, making ReS_2_ an excellent platform for investigating stacking as a tuning parameter for 2D materials. Furthermore, the optical anisotropy makes this material an interesting candidate for polarization-sensitive applications such as photodetectors and polarimetry.

## Introduction

Since the first mechanical exfoliation of graphene in 2004, much research effort has been invested in exploring the interesting physics of two-dimensional (2D) van der Waals (vdW) materials.^1^ Among these, the semiconducting transition metal dichalcogenides (TMDCs), such as MoS_2_ and WSe_2_, have drawn interest for their special optoelectronic properties.^2−4,5,6^ In their monolayer forms, dielectric screening of the free charges in these materials is reduced, enabling observation of stable Coulomb-bound quasi-particles of all varieties, including excitons, trions, and biexcitons at room temperature.^7−9,10^ These quasi-particles allow exploration of fundamental multiparticle physics, previously only accessible in more complex quantum-confined systems like quantum wells.^11^ In addition, in the monolayer limit, these excitons are highly susceptible to both internal and external tuning of their properties by strain, chemical doping, and electrical gating.^7,12−14^ While this sensitivity induces challenges in the consistent characterization of the intrinsic properties of these materials, it also presents exciting new opportunities for the application of these materials in tunable electrical and photonic devices.^15,16^

Symmetry breaking in the crystal structures of 2D materials allows for new phenomena and applications, such as linear polarization selectivity in black phosphorus (BP) and exciton-associated gate-tunability.^17^ Rhenium-based dichalcogenides, ReX_2_(X = S, Se), have received much attention as stable alternatives to BP, which often degrades in a few hours under ambient conditions.^18^ These rhenium compounds exhibit a triclinic crystal structure with a distorted octahedral coordination environment of the Re atoms.^19^ The reduced lattice symmetry results in linear polarization anisotropy of their optical properties.^20−22,23,24,25^ Remarkably, ReS_2_ exhibits clear excitonic resonances even in the bulk form. Its electronic band structure is uncharacteristically stable when going from monolayer to bulk,^19^ in stark contrast to the more common TMDCs such as MoS_2_ that exhibit abrupt transitions from direct to indirect band gap for monolayer and multilayer systems, respectively.^3^ This unique property of ReX_2_ materials has been termed “layer-decoupling” and is hypothesized to originate from a Peierls-like distortion of the crystal structure due to Re–Re intermetallic bonding.^19^ The accompanying strong excitonic confinement leads to high excitonic binding energies for bulk materials, in the range of 30–160 meV for the main excitons in ReS_2_.^26−28,29^ It has been argued that ReS_2_ has an indirect, or close-to-indirect band gap, due to its rather low photoluminescence quantum yield (PLQY) of order 10^–4^, an order of magnitude lower than that for other monolayer TMDCs,^30,31^ while calculations claim either direct or indirect.^33^ To add to the peculiarities, ReS_2_ has recently been shown to exist in two stable stacking variants, categorized as AA and AB, the latter with roughly half of a unit cell of lateral displacement between layers along the crystallographic *a*-axis.^32^ This previously unknown coexistence of stacking orders has contributed to the already existing discussions in the literature. These discussion typically centers around the wide range of reported exciton binding energies^26−28,29^ and the orientation of excitons with respect to the linear polarization angle.^25^ Coupled with the newly shown stacking parameter, the nature of excitons in ReS_2_ and their dependence on interlayer coupling remain unclear thus far.

Here, we combine polarization-resolved, low-temperature Raman, photoluminescence (PL) and reflectance spectroscopy to unravel the stacking-specific optical properties of ReS_2_ and connect them to the layer decoupling. We pinpoint the stacking order and crystal orientation using Raman spectroscopy and use polarization-resolved photoluminescence spectroscopy and differential reflectance spectroscopy to unambiguously determine the exciton alignment with respect to the crystal planes. We find very similar exciton polarizations in AB- and AA-stacked ReS_2_, but with a significant broadening of the excitonic peaks and strongly reduced signal intensity for AB. We attribute these modulated characteristics to a reduced exciton binding energy for AB-stacked ReS_2_, as compared with the AA variant. These results demonstrate that the optical properties of ReS_2_ are highly dependent on interlayer interactions and stacking order.

## Results

### Stacking Order Characterization

**Figure 1:**
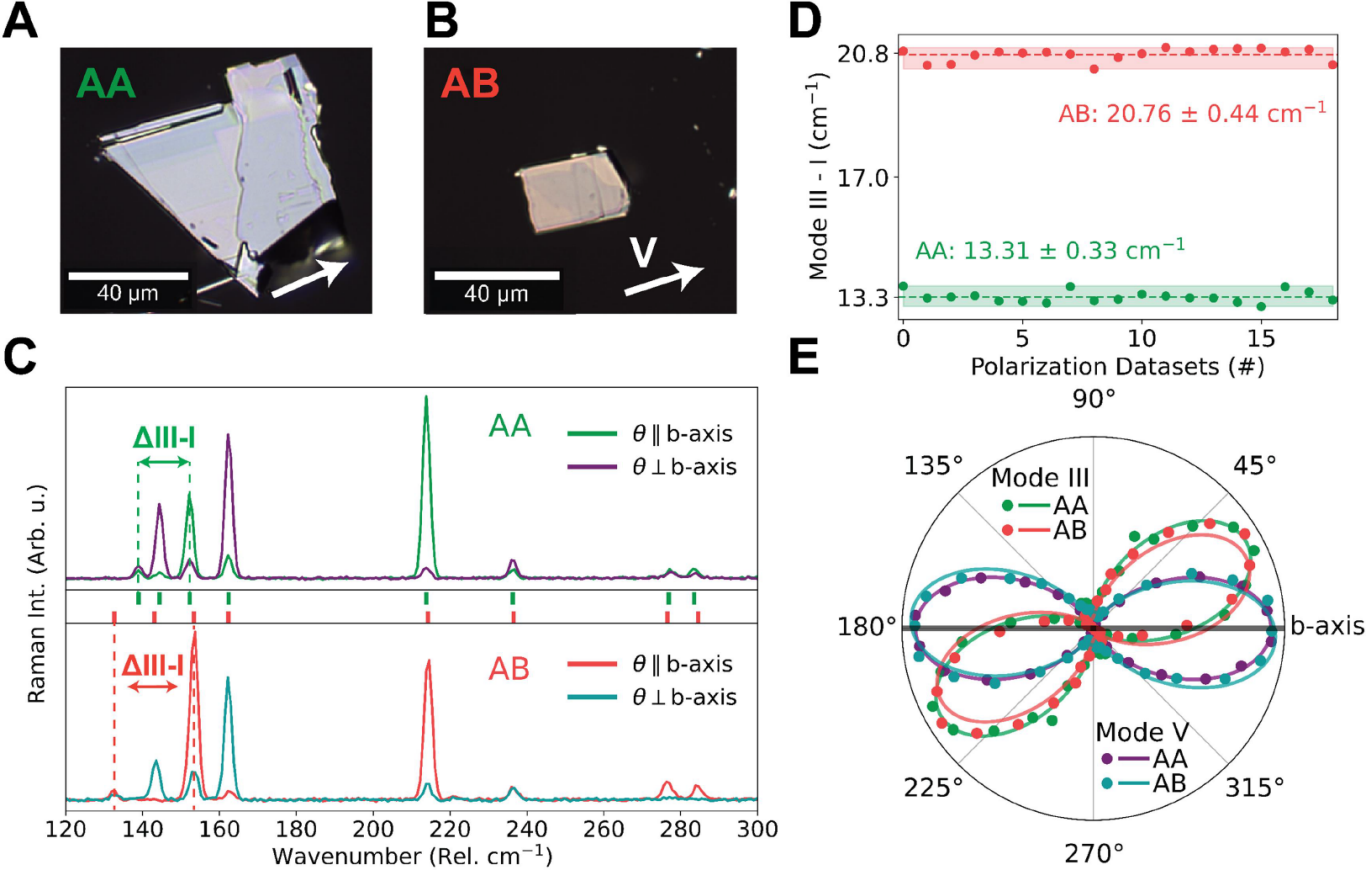
Crystal stacking order and orientation. (A, B) Optical microscope images of two bulk ReS_2_ flakes on a sapphire substrate. Arrows indicate the maximum-intensity polarization angle of Raman mode V, indicative of the *b*-axis direction. (C) Raman spectra of AA (top)- and AB (bottom)-stacked ReS_2_ at 83 K under 532 nm excitation. Spectra parallel and perpendicular to the *b*-axis are shown (see legend). Ticks between panels indicate peak energies. (D) Raman wavenumber difference between modes I and III for all data sets of linear polarizations. (E) Polar plot showing the polarization dependence of Raman mode III. Dots indicate the angle-dependent peak areas, and lines indicate the fits of these points using eq 1. Angles are given relative to the *b*-axis, as defined by the maximum intensity of the Raman mode V. The counterclockwise rotation direction from modes V to III indicates “upward” vertical orientation of the flakes.

Flakes of ReS_2_ are obtained by mechanical exfoliation from a commercially (HQ Graphene) obtained bulk crystal. Optical microscopy images of two exfoliated flakes are shown in Figure 1A,B. The flakes are bulklike, with lateral dimensions of a few tens of micrometers and thickness of a few 100 nm, as determined by atomic force microscopy measurements; see Figure S1. We use polarization-resolved Raman spectroscopy to determine their stacking order and vertical orientation. Low-temperature (83 K) Raman spectra of the flakes show the characteristic features for AA and AB stacking, as shown in Figure 1C. Here, we show spectra taken with linear polarizations parallel and perpendicular to the *b*-axis, as identified by the fifth mode in the Raman spectra.^24^ The energy difference between the first and third mode in the Raman spectra is characteristic of the stacking order.^32^ As shown in Figure 1D, the first flake shows a robust wavenumber difference between modes 1 and 3 of ∼13 cm^–1^, while the second flake shows a much larger shift of ∼21 cm^–1^, consistent with previous results on AA and AB stacked flakes.^32^ Also, this difference is significantly larger than the maximum variation in the individual peak energies. We hence associate the two flakes with AA-stacked and AB-stacked ReS_2_ which we refer to as “AA” and “AB” flakes, respectively. To identify the in- and out-of-plane crystal orientations, we perform Raman measurements for a full set of polarization angles (see Figure S2 for the full polarization-dependent data). The full Raman description of ReS_2_ is complex due to the material’s anisotropic character,^25,34−36,37^ and we discuss it in more detail in Section S2.1 of the Supporting Information. Here, we use the simplified, approximate form that captures the main polarization dependence of Raman intensities,
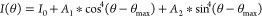
1where *I*(θ) is the area under a Raman peak at polarization angle θ, as determined from its Lorentzian fit, *I*_0_ the unpolarized contribution, *A*_1_ and *A*_2_ the weights of the cosine and sine functions, and θ_max_ the angle under which the Raman peak is maximum. The polarization-dependent Raman areas are shown together with their fits, as shown in Figure 1E, where angles are defined with respect to the orientation of the fifth mode (V), indicating the crystallographic *b*-axis.^24^ The third Raman mode (III) is also plotted and represents the orientation of the crystallographic *a*-axis.^36^ Clear overlap in the polarization dependence of the AA and AB flakes is observed. Furthermore, in both cases, a counterclockwise rotation leads from the fifth to the third mode, indicating an “upward” vertical orientation of the flakes.^25^ Polar plots of more Raman modes are shown in Figure S3. We hence conclude that we have flakes with AA and AB stacking orders, oriented in the same “upward” vertical direction and aligned with respect to their *b*-axis.

### Spectral Features of Excitons and Their Linear Polarization Dependence

**Figure 2:**
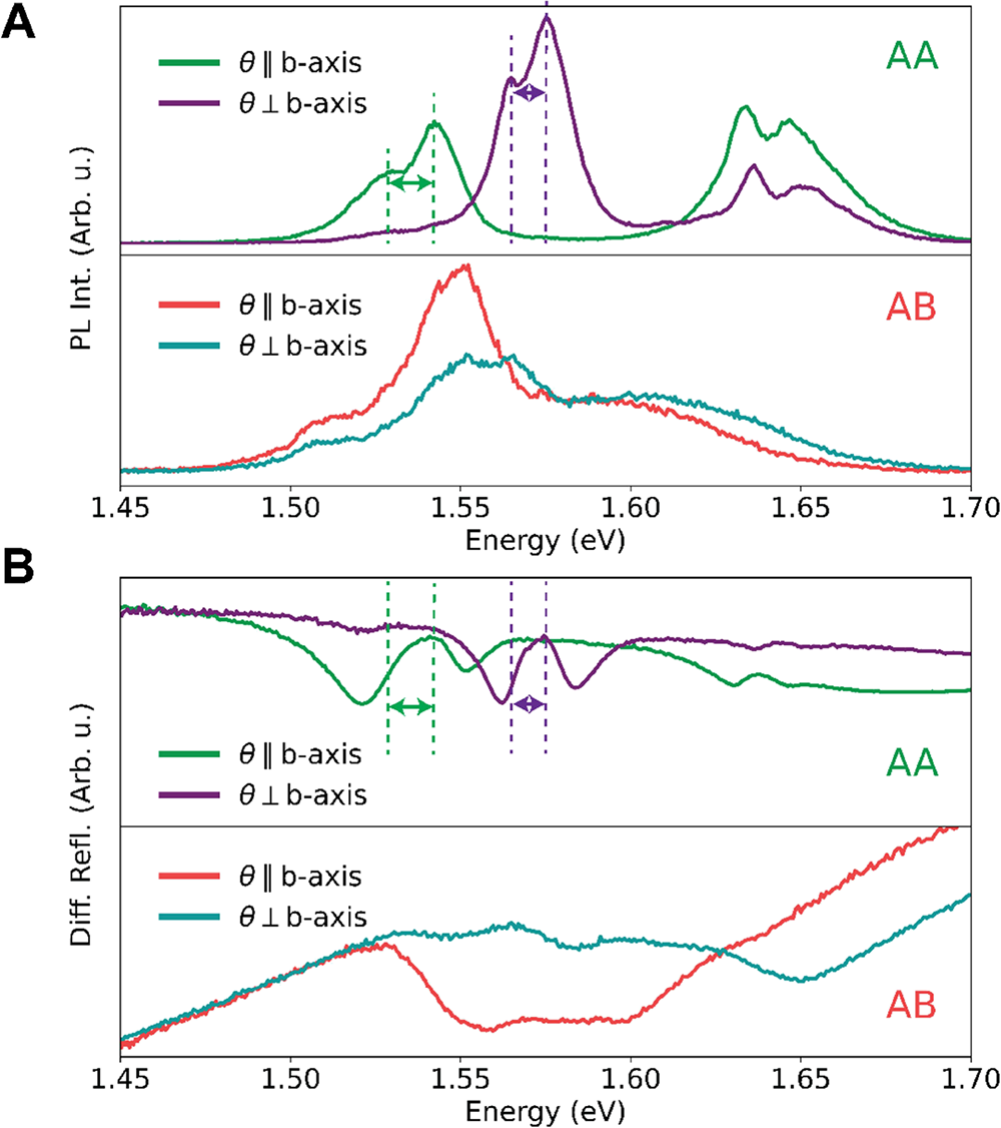
Excitonic features in photoluminescence and differential reflectance spectra. (A) Photoluminescence spectra, measured at 83K of both stacking orders for two polarization angles. In the top panel the AA stacking order shows 3 clear peak doublets at roughly 1.54, 1.57, and 1.65 eV. The bottom panel shows the same information for the AB stacking order; however, now, we observe less clearly defined peaks. This obscures the facile determination of peak origin. (B) Differential reflectance data, also measured at 83 K, for both stacking orders. The top panel clearly shows analogous behavior as the photoluminescence data with clearly defined peaks. For comparison, we have indicated the peak energies of the excitons from the PL spectra with dashed lines in the differential reflectance spectra.

To elucidate the excitonic properties of AA- and AB-stacked ReS_2_, we investigate their optical transitions using low-temperature photoluminescence (PL) and differential reflectance spectroscopy. PL spectra obtained for the two extreme polarization angles, parallel and perpendicular to the *b*-axis, are shown in Figure 2A. The full photoluminescence data sets are given in Figure S5A. The spectra of the AA flake are in good agreement with the literature:^27,29^ the two main features at 1.54 and 1.57 eV can be assigned to the two different exciton 1s states, which originate from the in-plane crystal anisotropy. Here, the double peaks for both excitons have been assigned to the neutral exciton and the lower-energy trion.^38,39^ Both have distinct polarization dependencies, while the higher-energy feature at around 1.65 eV is commonly ascribed to higher-excitation states of those excitons. The differential reflectance  spectra plotted in Figure 2B confirm these peak positions, showing clear features in agreement with the literature.^38,39^ In contrast, the PL spectra of the AB flake appear quite different, showing different spectral components and revealing a more isotropic response, in qualitative agreement with the room-temperature measurements of Zhou et al.^32^ The reflectance spectrum of the AB flake is more challenging to interpret. Its weak excitonic peaks suggest that the oscillator strength of the transitions is vastly reduced in AB-stacked flakes compared with AA-stacked flakes.

**Figure 3:**
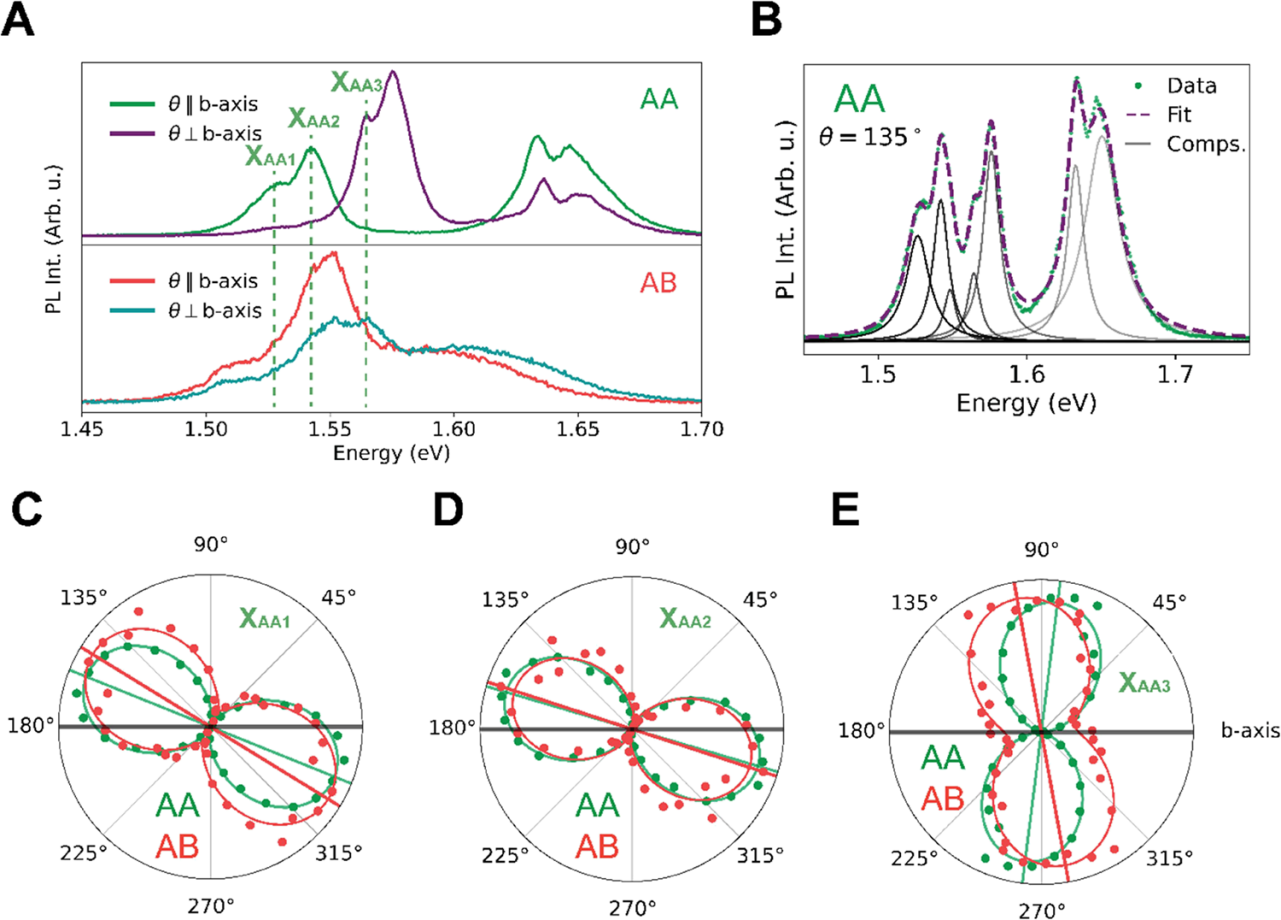
PL anisotropy of AA stacked ReS_2_. (A) Same photoluminescence spectra as plotted in Figure 2A, with 3 dashed lines indicating the three peaks that dominate in the spectra of the AA-stacked material. Our notation of X_AA1–3_ is used to avoid confusion with earlier interpretations in the literature. (B) Representative PL spectrum of the AA flake with 9 fitted subcomponents, of which 7 are clearly visible and two have a lower amplitude. (C–E) Polar plots of the integrated intensities (dots) of the fitted Lorentzian components from panel (B) for all photoluminescence spectra of both stacking orders. These integrated intensities are then fitted with the squared cosine behavior of the Malus law (dashed lines). These polar plots show that the polarization dependence for these spectral regions is preserved between the AA and AB stacking.

While different at the first glance, a closer look at the AA and AB spectra in Figure 2A reveals that they exhibit surprisingly similar excitonic features. Specifically, looking at the main peaks, it becomes clear that they are composed of similar subcomponents, apparent as shoulders, the main ones of the AA flake labeled X_AA1_ to X_AA3_ in Figure 3A. This is supported by the full polarization dependence of the peaks, shown in Figure S4, which reveals clear double lobes in the polar plots. In fact, the full spectral decomposition leads to 9 spectral components as can be seen by counting the peaks and shoulders shown in Figure S5. We, therefore, resolve the individual subcomponents of each peak and fit their polarization dependence separately. The polarization dependence of the components labeled X_AA1_ to X_AA3_ is compared for the AA and AB flake shown in panels C–E of Figure 3. Interestingly, although the corresponding spectral features are not easily recognized in the AB spectra, they nevertheless exhibit very similar polarization dependences, suggesting that they originate from similar excitonic states. We note that small deviations in alignment are possible due to small thickness-dependent variations of the fifth Raman mode orientation, which we use to define the *b*-axis.^40^ Indeed, fine resolution of the spectra reveals features at the same locations as the AA flake, distinguishable as shoulders in the AB spectrum. This is shown in Figure S5B, where some discussion on the origin of these peaks is also provided. This indicates that the PL peaks, clearly visible in AA, are also present in the AB flake, while their polarization dependence allows us to identify these peaks clearly. At the same time, the degree of polarization is reduced for the AB flake, particularly visible for the X_AA3_ peak, suggesting modification of the optical transition and hence the underlying excitonic state. We thus conclude that the characteristic differences between the AA and AB stacking are merely the different weights of the spectral components and the reduced degree of polarization of the AB flake.

**Figure 4:**
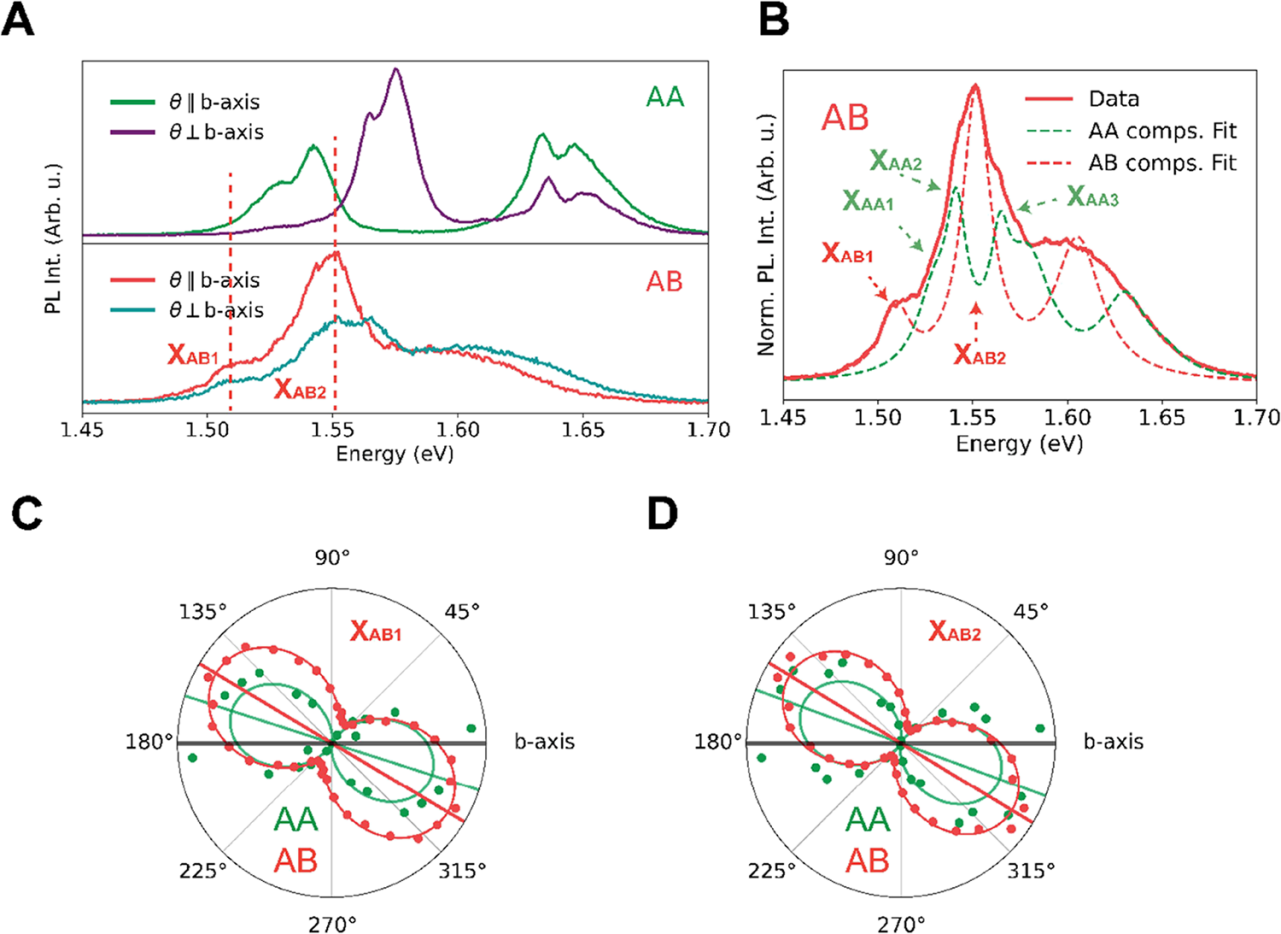
PL anisotropy of AB-stacked ReS_2_. (A) Same photoluminescence spectra as plotted in Figure 2A, similar to Figure 3A, we have plotted 2 dashed lines to indicate two dominant peaks in the spectra of the AB stacking order. Again, the notation of X_AB1,2_ is used to avoid confusion with earlier interpretations in the literature. (B) A plot showing the contributions (dashed) of various components to the total AB spectra (solid) for peaks most clearly visible in the AB spectrum (red dashed) and peaks most clearly visible in the AA spectrum (green dashed); arrows indicate components shown in polar plots. Note that the two dashed curves are artificially presented as separate components, i.e., they are plotted as two separate curves but come from a single comprehensive fit with one function. (C, D) Information displayed is as in the polar plots of Figure 3, now with the polarization dependence of two dominant peaks in the AB-spectra. Again, we see that the same spectral regions of the AA- and AB stacking orders have similar polarization-dependent behavior.

Vice versa, the similarity of the spectral features is also confirmed for the peaks dominant in the AB spectrum, labeled X_AB1_ and X_AB2_ in Figure 4A. Likewise, these spectral features seem absent in the AA spectrum, but can be identified upon closer inspection. Specifically, we identify an underlying peak at X_AB1_ from the asymmetric line shape of the main AA peak at 1.54 eV. The other peak X_AB2_ is visible as a shoulder in the PL spectra, as shown in Figure S5B. Full decomposition of the AB spectrum clearly shows the X_AA1_ to X_AA3_ components of the AA flake in addition to the hallmark X_AB1_ and X_AB2_ components of the AB flake shown in Figure 4B. We clearly see the presence of both AB (red dashed) and AA (green dashed) components, demonstrating again the contribution of both sets of features to the AB PL spectrum. The subfeatures X_AB1_ and X_AB2_ also exhibit very similar polarization dependence for AA and AB flakes, confirming their common origin, see Figure 4C,D. Again, the data suggest a slightly smaller polarization dependence of the AB flake.

The higher-energy peaks of the AA flake from 1.6 eV onward were previously identified as higher-excited states of the excitons with ground-state energies at 1.54 and 1.57 eV.^29^ If all these peaks and ground states are taken into account, then indeed their energies follow a Rydberg-like formula, resulting in an exciton binding energy of around 100 meV.^27,29^ In the AB flake, the high-energy features are also visible as broad shoulders yet are shifted to lower energies with respect to those of AA. Furthermore, their overall character has become less polarized. Applying a similar Rydberg-like analysis to the AB flake is difficult due to the significant peak broadening. Nevertheless, if we associate the broad feature in AB at around 1.61 eV with the higher-excited state, then we find that the energy difference between the ground and higher-excited exciton states is about 30 meV smaller than that in AA, which suggests an overall smaller exciton binding energy in AB compared with that in AA. The attribution of this broad feature to higher-excited states is supported by data of both polarization directions, showing consistent shifts of ground- and excited-state energies that are also consistent with those of AA: perpendicularly polarized excitons are always blue-shifted with respect to those with polarization parallel to the *b*-axis. Consistently, the lower energy difference between ground and excited states of both polarizations suggests smaller exciton binding energies in AB compared to AA.

Altogether, the similar polarization dependence of the photoluminescence spectra and the related peak positions indicate that we observe similar excitonic features in both stacking orders. However, the broadening of the spectral components and the less polarized response of the AB flake, as well as the redshift of the higher-excited states, highlight their clear differences. The latter is consistent with a reduced exciton binding energy of the AB flake, possibly caused by different interlayer interactions.

## Discussion

We measured linear-polarization-resolved photoluminescence spectra at 83 K to identify excitonic features of AA- and AB-stacked ReS_2_. While the similarity of peaks in the two flakes and their polarization dependence in principle could indicate some degree of mixed AA and AB stacking in a single flake, this is very unlikely, as the Raman spectra displayed in Figure 1 are strongly in favor of single-domain flakes. This interpretation is in agreement with Zhou et al.,^32^ which argued that mixed flakes are identifiable from Raman spectroscopy. We, therefore, conclude that the two flakes have distinct AA and AB stacking orders, and the spectra are representative thereof. The observed similarity of the two spectra then points to similarities in the underlying excitonic peak positions. Especially the comparison of the photoluminescence spectra integrated over all polarization angles (Figure S5B) confirms the similarity of spectral components of both stacking orders. This similarity supports the view that the excitonic properties in ReS_2_ are indeed to a large extent determined by the structure within the layers, which do not depend on their stacking order. The differences in the AA and AB spectra, specifically the reduction in exciton binding energy and peak broadening of the AB flake, then naturally indicate differences in the interlayer interactions, i.e., coupling, between the AA and AB layers.

**Figure 5:**
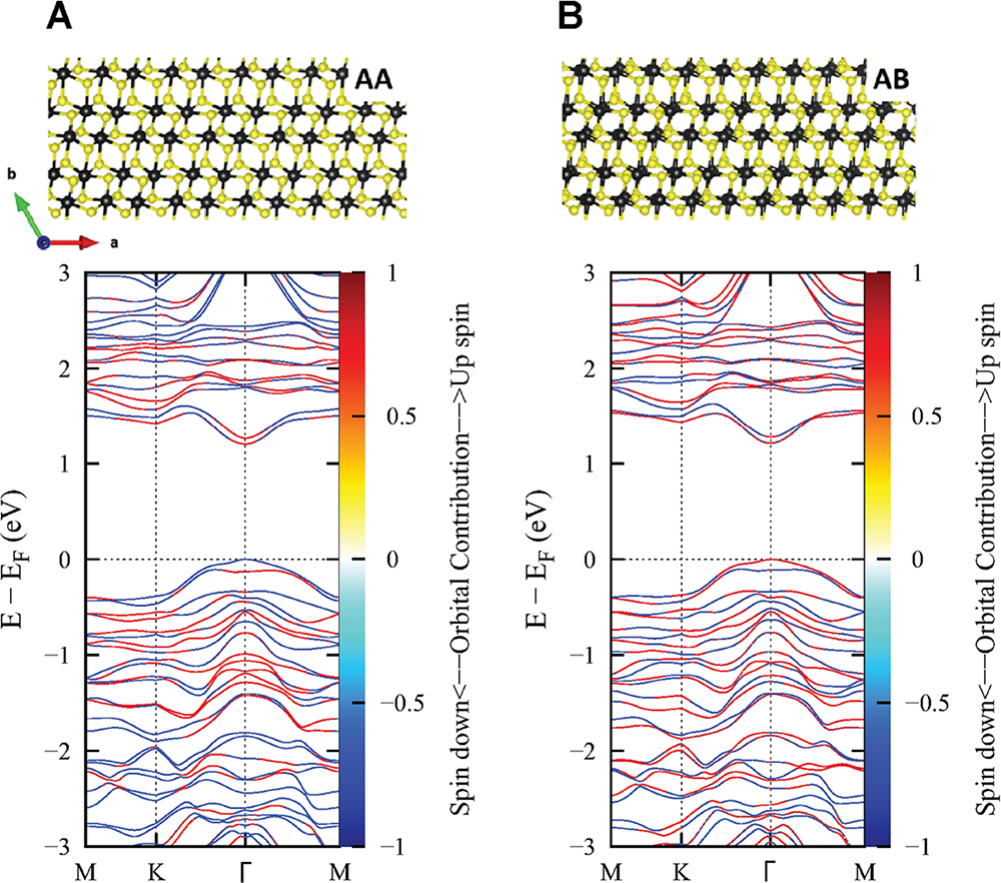
Electronic band structures. (Top) Crystal structures of the AA (A) and AB (B) bilayer systems. The first structure has the layers perfectly aligned on top of each other, while the latter structure is formed by displacing the second layer by roughly half of a unit cell along the crystallographic *a*-axis. This causes slightly different overlaps in atomic positions. (Bottom) Spin-projected band structure calculations of the respective systems were made using the optB86b-vdW functional including spin–orbit coupling. The color of the bands shows the spin-character, as indicated by the colormap on the right of the plots. Crucially, the most significant difference between AA- and AB-stacked ReS_2_ is the flip of the spin character of the valence bands, while the conduction bands are less affected. This change in the valence bands shows that the lowest transition is spin-dark in AA, but it becomes spin-bright in AB ReS_2_. The two valence bands have different dispersion, indicating different effective hole masses.

To investigate these interlayer interactions, we perform density functional theory (DFT) simulations of both stacking orders, with details described in Section S4 of the SI. We find that, curiously, there is no obvious difference between the stacking variants from the crystallochemical viewpoint (see Figures S6 and S7 and Table 1), for instance, in interatomic distances and angles, coordination environments of atoms across the van der Waals gap that could hint at increased interlayer directed bonding in one of the stackings. Moreover, dedicated calculations, taking into account spin–orbit coupling, show very similar band structures for both AA and AB, see Figure 5. Interestingly, however, these calculations reveal that the top of the valence bands is spin-split, with opposite spins for AA and AB, while the conduction band remains almost unchanged. This significant effect of spin–orbit coupling on the band structure is reminiscent of spin-dark states in group VI TMDCs,^41^ suggesting that these spin-projected states and the spin-flip character of the valence band could result in spin-forbidden transitions, effectively changing the optical band gap of AB- versus AA-ReS_2_.

Indeed, a recent study on ReS_2_ observed brightening of spin-dark states in AA-stacked ReS_2_ under magnetic field, although of several 10 meVs.^42^ Another work, by Dhara et al. reports weakly emitting states for AA-stacked ReS_2._^43^ Our DFT calculations confirm their suspicion that these weakly emitting states and the main excitons are spin-split. In AB stacking, these states become bright, with an associated lowering of the electronic band gap. This is independently confirmed when we look at the dispersion of the two valence bands to determine the effective masses of holes (see Table S2) in the optically allowed excitons. Optically allowed exciton states mainly consist of the red valence and conduction band states in Figure 5. Therefore, the optically allowed exciton in AA consists of a hole in the lower valence band, while in AB, the hole is in the upper valence band. The lower valence band is quite flat, leading to a large effective hole mass of the optically allowed exciton in AA. By contrast, the upper valence band has more dispersion, leading to a smaller effective hole mass in AB. The effective electron masses for AA and AB are similar. Taking this together, it turns out that the reduced mass of the optically allowed exciton in AB is smaller than that in AA; see Table S2 for the results of the DFT calculations. As the exciton binding energies scale linearly with the excitonic reduced mass according to the standard Rydberg-style formula, this means that the exciton binding energy of AB is smaller than that of AA. Quantitatively, from quadratic fits of the dispersions, we obtain reduced masses of μ = 0.365m_e_ and μ = 0.348m_e_, respectively, for AA and AB along the *M* – Γ – *M* path and μ = 0.406m_e_ and μ = 0.375m_e_ for the *K* – Γ – *K* direction (SI Section S4.4). This corroborates that the corresponding exciton binding energies are smaller for AB. This is indeed in qualitative agreement with our experimental findings of the reduced PL and reflectance intensities, as well as increased broadening of the exciton resonance of the AB stacking order, which are common indicators for exciton binding strength.^7^

We note that in our current approach, the DFT calculations are restricted to free-particle bands, in which exciton states are not adequately modeled. It is therefore conceivable that additional changes occur in the local dielectric environment, possibly modulating the screening of bound excitons.

In this regard, interesting connections may be drawn to the fractional dimensionality of the excitons. Originally introduced as a perturbation to the hydrogenic exciton to explain exciton binding energies in anisotropic, quantum-well structures that could not properly explained by pure 2D or 3D confinement,^44−46^ this concept was extended to explain exciton binding energies in 2D halide perovskites,^47^ as well as the gradual transition in exciton energy levels of MoS_2_ from monolayer to bulk.^48^ In ReS_2_, careful evaluation of the Rydberg fits showed that already in AA-stacked ReS_2_, the two differently polarized excitons could experience slightly different dimensionality in the plane.^44^ On a conceptual level, the more isotropic optical response we observe for AB-stacked ReS_2_ points to less confinement along certain in-plane crystal directions, possibly induced by a change in interlayer interactions upon stacking. This would suggest that not only do the ReS_2_ layers behave more bulk like but also within the plane, the excitons feel the crystal directions less in the AB-stacking than in the AA-stacking. Specifically, one of the excitons is located along the rhenium chains,^32^ and the lower anisotropy in the AB stacking indicates less of an alignment in this direction.

In other TMDCs, signs of altered interlayer interactions upon stacking have been experimentally observed in Raman, and PL and reflectance,^49−51^ some of which has been substantiated by theoretical calculations.^52^ However, in those cases, the differences in stacking orders are much smaller compared to the data we show here for bulk ReS_2_. For instance, two separate studies, on MoS_2_ and WSe_2_, looked at PL spectra of the respective bilayers.^49,51^ Interestingly, in both materials, a change in the energy differences of A and B excitons of about 20 meV was found, indicating that spin–orbit coupling effects are sensitive to stacking. In contrast, these studies on other TMDCs seem to show little change (∼<1 cm^–1^) in the shifts of main Raman peaks,^49−51^ while ReS_2_ shows easily observed shifts (∼7 cm^–1^), even at room temperature, upon stacking.

The reduction of PL intensity, peak broadening, and weakened reflection upon going from AA to AB stacking suggests a significant change in interlayer interactions. Our DFT simulations then indicate that this change in optical properties may be related to a flip in spin of the valence bands of AA and AB, which is accompanied by a change in effective hole masses, resulting in lower exciton binding energies for the AB stacking, as compared with AA. Finally, our extended simulations of the potential energy landscape of various ways of stacking successive layers suggest that other stacking orders could be experimentally realized in ReS_2_ (Figure S8), for which the interlayer coupling and optical properties of excitons could be further investigated.

## Conclusions

Using a combination of polarization-resolved spectroscopy techniques, we reveal surprisingly similar excitonic features in the two stacking orders of ReS_2_. However, their distinct modifications in terms of peak amplitude, peak broadening, and degree of polarization suggest different interlayer coupling in these stacking orders. Specifically, we attribute the peak broadening and reduced intensities in the AB stacking to increased interlayer interactions, consistent with a reduction in the exciton binding energies. In general, ReS_2_ is an interesting member of the 2D family of semiconductors due to its anisotropic crystal structure and its potential for polarization-sensitive applications, and the strong variations of excitonic properties due to stacking order suggest tunability of the excitonic states. Crucially, our results show that the polarization sensitivity can be retained, while the interlayer interactions are modulated with changes in the stacking order, making ReS_2_ an interesting candidate for investigating homo- and heterostructure properties in future studies.

## Methods

### Experiments

ReS_2_ flakes were mechanically exfoliated onto Si-SiO_2_ substrates from a commercially obtained bulk crystal (HQ-Graphene) by using pieces of blue Nitto tape (Nitto SPV-224PR-MJ). Exfoliation was performed 3–5 times, and bulk flakes were visually identified using a customized Witec 300 alpha R confocal microscope with a 50× magnification Zeiss objective (Objective LD EC “Epiplan-Neofluar” 50×/0.55 DIC M27). Atomic force microscopy data were taken using a Bruker Dimension FastScan system. Room-temperature measurements were performed on a piezo-scanning stage, while low-temperature measurements were performed in a Linkam cryostage (Linkam THMS350EV) that has a temperature range of about 77–475 K. Before low-temperature measurements were performed, the samples were baked in the cryostage at 473 K. Photoluminescence (PL) and Raman spectroscopies were performed using a diode-pumped 532 nm solid-state laser that was fiber-coupled into the Witec system via a reflective dichroic mirror that also acts as a laser filter on the detection side. Signals were collected via the same 50× Zeiss objective and out-coupled into a spectrograph (Witec UHTS 300Vis) connected to a CCD detector (Andor-Newton EMCCD). For the PL measurements, we used a 150 gr/mm (blaze 500 nm) grating, while the Raman measurements utilized a higher-resolution 1800 gr/mm (blaze 500 nm). The differential reflectance measurements were obtained using a broadband halogen lamp, correcting for the reflection of the substrate and electronic (dark) noise of the detector, while limiting the incident angles of the broadband light onto the samples by minimizing the aperture stop of the lamp. For all measurements shown in this work, the linear polarization angles of the polarizer and the analyzer (polarization filter) were kept parallel with respect to each other and thus rotated in the same way for successive measurements.

In the analysis, we find that all peaks in the Raman and photoluminescence spectra are reasonably well described by Lorentzian line shapes.

### Theoretical Modeling

All density functional theory (DFT) simulations were performed using the projector augmented wave (PAW) method implemented in the Vienna ab initio simulation package (VASP).^52−54^ To resolve the distinct stacking-order driven interlayer interactions of ReS_2_, two vdW dispersion corrections such as DFT-D2^55^ and DFT-D3,^56^ various exchange-correlation functionals in the nonlocal vdW-DF family such as optPBE, optB86b,^57^ optB88,^58^ Grimme’s PBE-DF2,^59^ and the hybrid functional HSE06^60^ were used in this study. The integration in the Brillouin zone was employed using the Monkhorst–Pack scheme^61^ (11 × 11 × 1) with an energy cutoff of 500 eV. The convergence threshold for the self-consistent field calculations was set to 10^–5^ eV per cell, and the geometrical structures were fully optimized until the Hellmann–Feynman forces acting on atoms were less than 0.01 eV Å^–1^.^62^ For more details about the simulation and the comparison of various functionals, see SI.

## Supporting Information Available

Atomic force microscopy measurements; polar plots Raman and PL; multiple flakes data; and density functional theory calculations (PDF)

## References

[ref1] Novoselov K. S.; Geim A. K.; Morozov S. V.; Jiang D. E.; Zhang Y.; Dubonos S. V.; Grigorieva I. V.; Firsov A. A. Electric Field Effect in Atomically Thin Carbon Films. Science 2004, 306 (5696), 666–669. 10.1126/science.1102896. 15499015

[ref2] Xia F.; Wang H.; Xiao D.; Dubey M.; Ramasubramaniam A. Two-Dimensional Material Nanophotonics. Nat. Photonics 2014, 8 (12), 899–907. 10.1038/nphoton.2014.271.

[ref3] Splendiani A.; Sun L.; Zhang Y.; Li T.; Kim J.; Chim C. Y.; Galli G.; Wang F. Emerging Photoluminescence in Monolayer MoS2. Nano Lett. 2010, 10 (4), 1271–1275. 10.1021/nl903868w. 20229981

[ref4] Mak K. F.; Lee C.; Hone J.; Shan J.; Heinz T. F. Atomically Thin MoS 2: a New Direct-Gap Semiconductor. Phys. Rev. Lett. 2010, 105 (13), 136805 10.1103/PhysRevLett.105.136805. 21230799

[ref5] Ramakrishna Matte H. S. S.; Gomathi A.; Manna A. K.; Late D. J.; Datta R.; Pati S. K.; Rao C. N. R. MoS2 and WS2 Analogues of Graphene. Angew. Chem., Int. Ed. 2010, 49 (24), 4059–4062. 10.1002/anie.201000009. 20425874

[ref6] Mak K. F.; Shan J. Photonics and Optoelectronics of 2D Semiconductor Transition Metal Dichalcogenides. Nat. Photonics 2016, 10 (4), 216–226. 10.1038/nphoton.2015.282.

[ref7] Wang G.; Chernikov A.; Glazov M. M.; Heinz T. F.; Marie X.; Amand T.; Urbaszek B. Colloquium: Excitons in Atomically Thin Transition Metal Dichalcogenides. Rev. Mod. Phys. 2018, 90 (2), 021001 10.1103/RevModPhys.90.021001.

[ref8] Mak K. F.; He K.; Lee C.; Lee G. H.; Hone J.; Heinz T. F.; Shan J. Tightly Bound Trions in Monolayer MoS 2. Nat. Mater. 2013, 12 (3), 207–211. 10.1038/nmat3505. 23202371

[ref9] Ross J. S.; Wu S.; Yu H.; Ghimire N. J.; Jones A. M.; Aivazian G.; Yan J.; Mandrus D. G.; Xiao D.; Yao W.; Xu X. Electrical Control of Neutral and Charged Excitons in a Monolayer Semiconductor. Nat. Commun. 2013, 4 (1), 1474 10.1038/ncomms2498. 23403575

[ref10] Sie E. J.; Frenzel A. J.; Lee Y. H.; Kong J.; Gedik N. Intervalley Biexcitons and Many-Body Effects in Monolayer MoS 2. Phys. Rev. B 2015, 92 (12), 125417 10.1103/PhysRevB.92.125417.

[ref11] Weisbuch C.; Benisty H.; Houdré R. Overview of Fundamentals and Applications of Electrons, Excitons and Photons in Confined Structures. J. Lumin. 2000, 85 (4), 271–293. 10.1016/S0022-2313(99)00194-5.

[ref12] Amani M.; Lien D. H.; Kiriya D.; Xiao J.; Azcatl A.; Noh J.; Madhvapathy S. R.; Addou R.; Santosh K. C.; Dubey M.; Cho K. Near-Unity Photoluminescence Quantum Yield in MoS2. Science 2015, 350 (6264), 1065–1068. 10.1126/science.aad2114. 26612948

[ref13] Lien D. H.; Uddin S. Z.; Yeh M.; Amani M.; Kim H.; Ager J. W.; Yablonovitch E.; Javey A. Electrical Suppression of All Nonradiative Recombination Pathways in Monolayer Semiconductors. Science 2019, 364 (6439), 468–471. 10.1126/science.aaw8053. 31048488

[ref14] Aslan O. B.; Deng M.; Heinz T. F. Strain Tuning of Excitons in Monolayer WSe 2. Phys. Rev. B 2018, 98 (11), 115308 10.1103/PhysRevB.98.115308.

[ref15] Lynch J.; Guarneri L.; Jariwala D.; van de Groep J. Exciton Resonances for Atomically-Thin Optics. J. Appl. Phys. 2022, 132 (9), 091102 10.1063/5.0101317.

[ref16] Jariwala D.; Sangwan V. K.; Lauhon L. J.; Marks T. J.; Hersam M. C. Emerging Device Applications for Semiconducting Two-Dimensional Transition Metal Dichalcogenides. ACS Nano 2014, 8 (2), 1102–1120. 10.1021/nn500064s. 24476095

[ref17] Biswas S.; Whitney W. S.; Grajower M. Y.; Watanabe K.; Taniguchi T.; Bechtel H. A.; Rossman G. R.; Atwater H. A. Tunable Intraband Optical Conductivity and Polarization-Dependent Epsilon-Near-Zero Behavior in Black Phosphorus. Sci. Adv. 2021, 7 (2), eabd4623 10.1126/sciadv.abd4623. 33523990 PMC7793587

[ref18] Castellanos-Gomez A.; Vicarelli L.; Prada E.; Island J. O.; Narasimha-Acharya K. L.; Blanter S. I.; Groenendijk D. J.; Buscema M.; Steele G. A.; Alvarez J. V.; Zandbergen H. W. Isolation and Characterization of Few-Layer Black Phosphorus. 2D Mater. 2014, 1 (2), 025001 10.1088/2053-1583/1/2/025001.

[ref19] Tongay S.; Sahin H.; Ko C.; Luce A.; Fan W.; Liu K.; Zhou J.; Huang Y. S.; Ho C. H.; Yan J.; Ogletree D. F. Monolayer Behaviour in Bulk ReS2 Due to Electronic and Vibrational Decoupling. Nat. Commun. 2014, 5 (1), 3252 10.1038/ncomms4252. 24500082

[ref20] Webb J. L.; Hart L. S.; Wolverson D.; Chen C.; Avila J.; Asensio M. C. Electronic Band Structure of ReS2 by High-Resolution Angle-Resolved Photoemission Spectroscopy. Phys. Rev. B 2017, 96 (11), 115205 10.1103/PhysRevB.96.115205. PMC5506037 28698655

[ref21] Gehlmann M.; Aguilera I.; Bihlmayer G.; Nemšák S.; Nagler P.; Gospodaric P.; Zamborlini G.; Eschbach M.; Feyer V.; Kronast F.; Młyńczak E. Direct Observation of the Band Gap Transition in Atomically Thin ReS2. Nano Lett. 2017, 17 (9), 5187–5192. 10.1021/acs.nanolett.7b00627. 28759250

[ref22] Biswas D.; Ganose A. M.; Yano R.; Riley J. M.; Bawden L.; Clark O. J.; Feng J.; Collins-Mcintyre L.; Sajjad M. T.; Meevasana W.; Kim T. K. Narrow-Band Anisotropic Electronic Structure of ReS2. Phys. Rev. B 2017, 96 (8), 085205 10.1103/PhysRevB.96.085205.

[ref23] Aslan O. B.; Chenet D. A.; Van Der Zande A. M.; Hone J. C.; Heinz T. F. Linearly Polarized Excitons in Single-And Few-Layer ReS2 Crystals. ACS Photonics 2016, 3 (1), 96–101. 10.1021/acsphotonics.5b00486.

[ref24] Chenet D. A.; Aslan O. B.; Huang P. Y.; Fan C.; Van Der Zande A. M.; Heinz T. F.; Hone J. C. In-Plane Anisotropy in Mono-And Few-Layer ReS2 Probed by Raman Spectroscopy and Scanning Transmission Electron Microscopy. Nano Lett. 2015, 15 (9), 5667–5672. 10.1021/acs.nanolett.5b00910. 26280493

[ref25] Hart L.; Dale S.; Hoye S.; Webb J. L.; Wolverson D. Rhenium Dichalcogenides: Layered Semiconductors with Two Vertical Orientations. Nano Lett. 2016, 16 (2), 1381–1386. 10.1021/acs.nanolett.5b04838. 26799768

[ref26] Ho C. H.; Liao P. C.; Huang Y. S.; Tiong K. K. Temperature Dependence of Energies and Broadening Parameters of the Band-Edge Excitons of ReS2 and ReSe2. Phys. Rev. B 1997, 55 (23), 15608 10.1103/PhysRevB.55.15608.

[ref27] Ho C. H.; Liu Z. Z. Complete-Series Excitonic Dipole Emissions in Few Layer ReS2 and ReSe2 Observed by Polarized Photoluminescence Spectroscopy. Nano Energy 2019, 56, 641–650. 10.1016/j.nanoen.2018.12.014.

[ref28] Ho C. H.; Yen P. C.; Huang Y. S.; Tiong K. K. Photoreflectance Study of the Excitonic Transitions of Rhenium Disulphide Layer Compounds. Phys. Rev. B 2002, 66, 245207 10.1103/PhysRevB.66.245207.

[ref29] Jadczak J.; Kutrowska-Girzycka J.; Smoleński T.; Kossacki P.; Huang Y. S.; Bryja L. Exciton Binding Energy and Hydrogenic Rydberg Series in Layered ReS2. Sci. Rep. 2019, 9 (1), 1578 10.1038/s41598-018-37655-8. 30733485 PMC6367321

[ref30] Chernikov A.; Berkelbach T. C.; Hill H. M.; Rigosi A.; Li Y.; Aslan O. B.; Reichman D. R.; Hybertsen M. S.; Heinz T. F. Exciton Binding Energy and Nonhydrogenic Rydberg Series in Monolayer WS2. Phys. Rev. Lett. 2014, 113, 076802 10.1103/PhysRevLett.113.076802. 25170725

[ref31] Mohamed N. B.; Shinokita K.; Wang X.; Lim H. E.; Tan D.; Miyauchi Y.; Matsuda K. Photoluminescence Quantum Yields for Atomically Thin-Layered ReS2: Identification of Indirect-Bandgap Semiconductors. Appl. Phys. Lett. 2018, 113 (12), 121112 10.1063/1.5037116.

[ref32] Zhou Y.; Maity N.; Rai A.; Juneja R.; Meng X.; Roy A.; Zhang Y.; Xu X.; Lin J. F.; Banerjee S. K.; Singh A. K. Stacking-Order-Driven Optical Properties and Carrier Dynamics in ReS2. Adv. Mater. 2020, 32 (22), 1908311 10.1002/adma.201908311. 32329148

[ref33] Bae S.; Sim S. Anisotropic Excitons in 2D Rhenium Dichalcogenides: a Mini-Review. J. Korean Phys. Soc. 2022, 81, 532–548. 10.1007/s40042-022-00401-5.

[ref34] Kranert C.; Sturm C.; Schmidt-Grund R.; Grundmann M. Raman Tensor Formalism for Optically Anisotropic Crystals. Phys. Rev. Lett. 2016, 116 (12), 127401 10.1103/PhysRevLett.116.127401. 27058099

[ref35] Zhang S.; Mao N.; Zhang N.; Wu J.; Tong L.; Zhang J. Anomalous Polarized Raman Scattering and Large Circular Intensity Differential in Layered Triclinic ReS2. ACS Nano 2017, 11 (10), 10366–10372. 10.1021/acsnano.7b05321. 28992402

[ref36] aMcCreary A.; Simpson J. R.; Wang Y.; Rhodes D.; Fujisawa K.; Balicas L.; Dubey M.; Crespi V. H.; Terrones M.; Hight Walker A. R. Intricate Resonant Raman Response in Anisotropic ReS2. Nano Lett. 2017, 17 (10), 5897–5907. 10.1021/acs.nanolett.7b01463. 28820602

[ref37] Choi Y.; Kim K.; Lim S. Y.; Kim J.; Park J. M.; Kim J. H.; Cheong H. Complete determination of the crystallographic orientation of ReX2 (X= S, Se) by polarized Raman spectroscopy. Nanoscale Horiz. 2020, 5 (2), 308–315. 10.1039/C9NH00487D.

[ref38] Wang X.; Shinokita K.; Matsuda K. Radiative Lifetime and Dynamics of Trions in Few-Layered ReS2. Appl. Phys. Lett. 2021, 119 (11), 113103 10.1063/5.0059198.

[ref39] Wang X.; Shinokita K.; Miyauchi Y.; Cuong N. T.; Okada S.; Matsuda K. Experimental Evidence of Anisotropic and Stable Charged Excitons (Trions) in Atomically Thin 2D ReS2. Adv. Funct. Mater. 2019, 29 (51), 1905961 10.1002/adfm.201905961.

[ref40] Wu R.; Qi M.; Zhao Q.; Huang Y.; Zhou Y.; Xu X. Anomalous Polarization Pattern Evolution of Raman Modes in Few-Layer ReS2 by Angle-Resolved Polarized Raman Spectroscopy. Nanoscale 2022, 14 (5), 1896–1905. 10.1039/D1NR06733H. 35044412

[ref41] Echeverry J. P.; Urbaszek B.; Amand T.; Marie X.; Gerber I. C. Splitting Between Bright and Dark Excitons in Transition Metal Dichalcogenide Monolayers. Phys. Rev. B 2016, 93, 121107 10.1103/PhysRevB.93.121107.

[ref42] Kapuściński P.; Dzian J.; Slobodeniuk A. O.; Rodríguez-Fernández C.; Jadczak J.; Bryja L.; Faugeras C.; Baso D. M.; Potemski M. Exchange-Split Multiple Rydberg Series of Excitons in Anisotropic Quasi Two-Dimensional ReS2. 2D Mater. 2022, 9 (4), 045005 10.1088/2053-1583/ac7880.

[ref43] Dhara A.; Chakrabarty D.; Das P.; Pattanayak A. K.; Paul S.; Mukherjee S.; Dhara S. Additional excitonic features and momentum-dark states in ReS2. Phys. Rev. B 2020, 102, 161404 10.1103/PhysRevB.102.161404.

[ref44] He X. F. Excitons in Anisotropic Solids: The Model of Fractional-Dimensional Space. Phys. Rev. B 1991, 43 (3), 2063 10.1103/PhysRevB.43.2063. 9997475

[ref45] Christol P.; Lefebvre P.; Mathieu H. Fractional-Dimensional Calculation of Exciton Binding Energies in Semiconductor Quantum Wells and Quantum-Well Wires. J. Appl. Phys. 1993, 74 (9), 5626–5637. 10.1063/1.354224.

[ref46] Lefebvre P.; Christol P.; Mathieu H. Unified Formulation of Excitonic Absorption Spectra of Semiconductor Quantum Wells, Superlattices, and Quantum Wires. Phys. Rev. B 1993, 48 (23), 17308 10.1103/PhysRevB.48.17308. 10008340

[ref47] Blancon J. C.; Stier A. V.; Tsai H.; Nie W.; Stoumpos C. C.; Traore B.; Pedesseau L.; Kepenekian M.; Katsutani F.; Noe G. T.; Kono J.; Tretiak S.; Crooker S. A.; Katan C.; Kanatzidis M. G.; Crochet J. J.; Even J.; Mohite A. D. Scaling Law for Excitons in 2D Perovskite Quantum Wells. Nat. Commun. 2018, 9 (1), 2254 10.1038/s41467-018-04659-x. 29884900 PMC5993799

[ref48] Jia G. Y.; Liu Y.; Gong J. Y.; Lei D. Y.; Wang D. L.; Huang Z. X. Excitonic Quantum Confinement Modified Optical Conductivity of Monolayer and Few-Layered MoS2. J. Mater. Chem. C 2016, 4 (37), 8822–8828. 10.1039/C6TC02502A.

[ref49] Shinde S. M.; Dhakal K. P.; Chen X.; Yun W. S.; Lee J.; Kim H.; Ahn J. H. Stacking-Controllable Interlayer Coupling and Symmetric Configuration of Multilayered MoS2. NPG Asia Mater. 2018, 10 (2), e468 10.1038/am.2017.226.

[ref50] Liu K.; Zhang L.; Cao T.; Jin C.; Qiu D.; Zhou Q.; Zettl A.; Yang P.; Louie S. G.; Wang F. Evolution of Interlayer Coupling in Twisted Molybdenum Disulfide Bilayers. Nat. Commun. 2014, 5 (1), 4966 10.1038/ncomms5966. 25233054

[ref51] McCreary K. M.; Phillips M.; Chuang H. J.; Wickramaratne D.; Rosenberger M.; Hellberg C. S.; Jonker B. T. Stacking-Dependent Optical Properties in Bilayer WSe2. Nanoscale 2021, 14 (1), 147–156. 10.1039/D1NR06119D. 34904621

[ref52] He J.; Hummer K.; Franchini C. Stacking Effects on the Electronic and Optical Properties of Bilayer Transition Metal Dichalcogenides MoS2, MoSe2, WS2, and WSe2. Phys. Rev. B 2014, 89, 075409 10.1103/PhysRevB.89.075409.

[ref53] Kresse G.; Hafner J. Ab Initio Molecular-Dynamics Simulation of the Liquid-Metal–Amorphous-Semiconductor Transition in Germanium. Phys. Rev. B 1994, 49 (20), 14251 10.1103/PhysRevB.49.14251. 10010505

[ref54] Kresse G.; Joubert D. From Ultrasoft Pseudopotentials to the Projector Augmented-Wave Method. Phys. Rev. B 1999, 59 (3), 1758 10.1103/PhysRevB.59.1758.

[ref55] Kresse G.; Furthmüller J. Efficiency of Ab-Initio Total Energy Calculations for Metals and Semiconductors Using a Plane-Wave Basis Set. Comput. Mater. Sci. 1996, 6 (1), 15–50. 10.1016/0927-0256(96)00008-0.

[ref56] Bucko T.; Hafner J.; Lebegue S.; Angyan J. G. Improved Description of the Structure of Molecular and Layered Crystals: Ab Initio DFT Calculations with Van Der Waals Corrections. J. Phys. Chem. A 2010, 114 (43), 11814–11824. 10.1021/jp106469x. 20923175

[ref57] Grimme S.; Antony J.; Ehrlich S.; Krieg H. A Consistent and Accurate Ab Initio Parametrization of Density Functional Dispersion Correction (DFT-D) for the 94 Elements H-Pu. J. Chem. Phys. 2010, 132 (15), 154104 10.1063/1.3382344. 20423165

[ref58] Klimeš J.; Bowler D. R.; Michaelides A. Van Der Waals Density Functionals Applied to Solids. Phys. Rev. B 2011, 83, 195131 10.1103/PhysRevB.83.195131.

[ref59] Klimeš J.; Bowler D. R.; Michaelides A. Chemical Accuracy for the Van Der Waals Density Functional. J. Phys.: Condens. Matter 2010, 22 (2), 022201 10.1088/0953-8984/22/2/022201. 21386245

[ref60] Lee K.; Murray É.D.; Kong L.; Lundqvist B. I.; Langreth D. C. Higher-Accuracy Van Der Waals Density Functional. Phys. Rev. B 2010, 82, 081101 10.1103/PhysRevB.82.081101.

[ref61] Monkhorst H. J.; Pack J. D. Special Points for Brillouin-Zone integrations. Phys. Rev. B 1976, 13 (12), 5188 10.1103/PhysRevB.13.5188.

[ref62] Krukau A. V.; Vydrov O. A.; Izmaylov A. F.; Scuseria G. E. Influence of the Exchange Screening Parameter on the Performance of Screened Hybrid Functionals. J. Chem. Phys. 2006, 125 (22), 224106 10.1063/1.2404663. 17176133

