# Supplementary material for: Stacking-Order-Dependent Excitonic Properties Reveal Interlayer Interactions in Bulk ReS2
Source: ACS Photonics. 2023 Aug 16;10(9):3115–23. doi: 10.1021/acsphotonics.3c00477 (PMC10515696; doi:10.1021/acsphotonics.3c00477)
Supplement: Supplementary file 1 — ph3c00477_si_001.pdf [file ph3c00477_si_001.pdf]

Supplementary Information for article

"Stacking-order-dependent Excitonic Properties Reveal Interlayer Interactions in Bulk ReS_2"

M. van der Laan[1], E. Heemskerk[1], F. Kienhuis[1], N. Diepeveen[1], D. Poonia[2], S. Kinge[2,3], M.T. Dang[4], V.A. Dinh[5], L.D.A. Siebbeles[2], A. Isaeva[1], J. van de Groep[1] and P. Schall[1]

Affiliations

[1] Institute of Physics, University of Amsterdam, Science Park 904, 1098 XH Amsterdam, The Netherlands
[2] Optoelectronic Materials Section, Department of Chemical Engineering, Delft University of Technology 2629 HZ Delft, The Netherlands
[3] Materials Research & Development, Toyota Motor Europe, B1930 Zaventem, Belgium
[4] School of Education, Can Tho University, 3-2 Road, Can Tho City 900000, Vietnam
[5] Department of Precision Engineering, Graduate School of Engineering, Osaka University, 2-1 Yamadaoka, Suita, Osaka 565-0871, Japan

Email: p.schall@uva.nl

Contents

1. AFM images and scans
2. Supplementary Raman
3. Supplementary photoluminescence figures
4. Density Functional Theory simulations
5. References

15 pages
9 figures
4 equations
2 tables

1. AFM images and scans

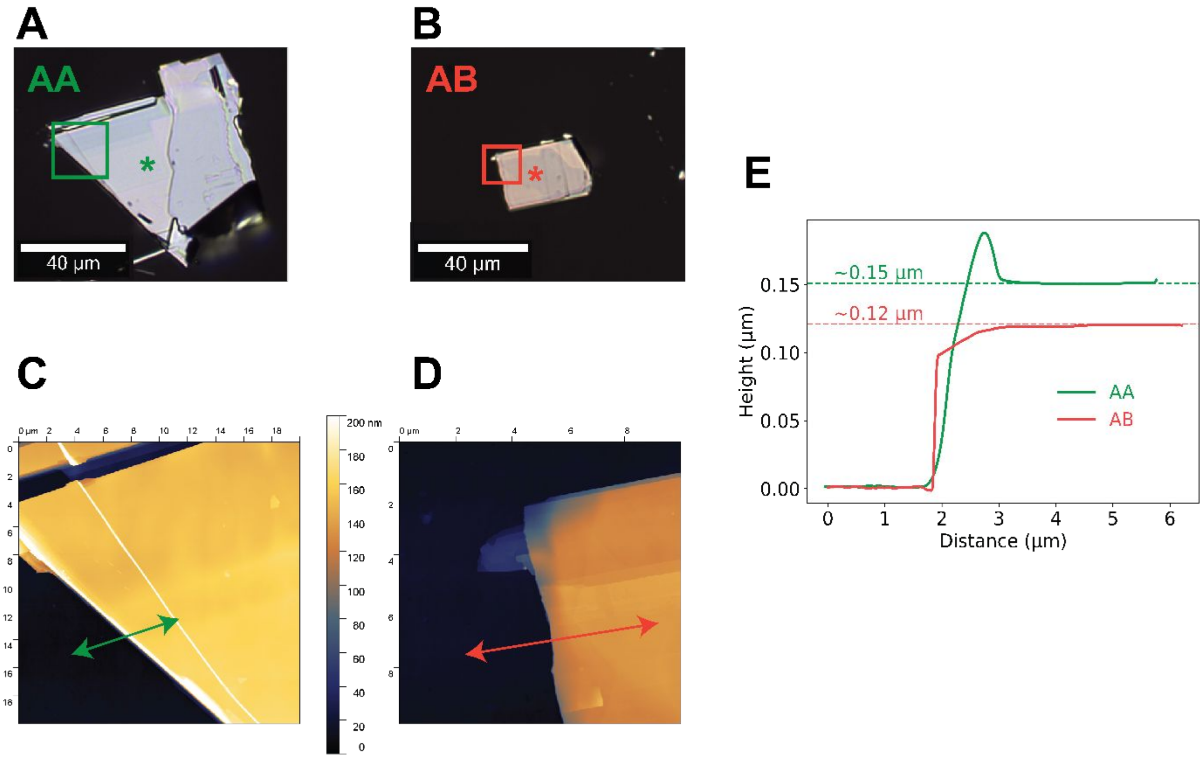

Figure S1. Atomic Force Microscopy (AFM) scans and data
A,B) Optical images of the two flakes. Coloured boxes indicate the areas of the AFM scans and the '*' symbols roughly indicate the measurement spot for the Raman, PL and reflection data shown in the main text.
C,D) Maps of the AFM data. Colours indicate the vertical height. Both maps have the same colour scale from 0 to 200 micrometer. The coloured arrows indicate the chosen profile lines.
E) Extracted profiles of the AFM data. Solid lines indicate the profiles of the AA (green) and AB (red) flakes. Background has been scaled to zero, and the onsets, around 2 micron on the horizontal axis, of the flakes have been shifted such that the profiles overlap. Dashed lines are a guide to the eye and the height of the flakes are roughly 150nm and 120nm for the AA and AB flakes respectively. This very roughly corresponds to a thickness of around 200 monolayers, clearly bulk.

2. Supplementary Raman

2.1 Explanation of Raman fitting function

The full Raman description of ReS_2 is complex due to the material's anisotropic character.[1-5] Briefly, the action of optical plates, such as polarizers, can be represented as linear transformations of the incident electric field. Similarly, Raman scattering can be described in tensor form. The elements of these matrices are determined by the bulk crystal symmetry and an additional complex phase is introduced to account for the anisotropic absorption.[2] The full description for the Raman modes of ReS_2 is then connected to experiments by applying the Raman tensor to the polarization directions of incident and detected light. In our case, the incident and detected angles were kept equal, i.e. parallel configuration. The intensity of each individual Raman mode can then be described by

$$\begin{aligned} I(\theta) = & A_1^2 * \cos^4(\theta-\theta_{max}) + A_2^2 * \sin^4(\theta-\theta_{max}) \\ & + A_3^2 * \cos^2(\theta-\theta_{max}) * \sin^2(\theta-\theta_{max}) \\ & + A_1 * A_2 * \cos(2*\phi) * \cos^2(\theta-\theta_{max}) * \sin^2(\theta-\theta_{max}) \\ & + A_1 * A_3 * \cos(\phi) * \cos^3(\theta-\theta_{max}) * \sin^1(\theta-\theta_{max}) \\ & + A_2 * A_3 * \cos(\phi) * \cos^1(\theta-\theta_{max}) * \sin^3(\theta-\theta_{max}) \end{aligned} \quad \text{(S1)}$$

Here, A_i is the amplitude of each relevant matrix element, θ is the polarization angle with respect to the laboratory axes, θ_{max} is the angle of polarization under which the intensity of the Raman mode is maximum, and ϕ represents the phase of each Raman tensor element. The phase factor is directly related to anisotropic absorption and as such is experimentally connected to both the excitation wavelength and thickness of the sample. As our experiments were carried out under identical conditions, and the contribution of terms with the phase factor seemed unnecessary to provide good fits, we decided to leave them out. This is a somewhat coincidental interplay of circumstances as vastly different thicknesses or different excitation wavelengths might have resulted in stronger contributions of these terms, as shown by Zhang et al.[2] Furthermore, as pointed out by Kranert et al., off-diagonal terms, A_3 in our case, of Raman tensors are generally at least one order of magnitude weaker than the diagonal terms.[1] Therefore, in our fitting procedure we have approximated the Raman response with just the first two terms, and parameterized the resulting function as

$$I(\theta) = I_0 + A_1 * \cos^4(\theta-\theta_{max}) + A_2 * \sin^4(\theta-\theta_{max}), \quad \text{(S2)}$$

where $I(\theta)$ is the area under a Raman peak at polarization angle θ, as determined from its Lorentzian fit, I_0 the unpolarized contribution, A_1 and A_2 the weights of the cosine and sine functions and θ_{max} the angle under which the Raman peak is maximum.

Equation S2 is the fitting function used for all Raman fits, as discussed in the main text.

2.2 Supplementary Raman figures

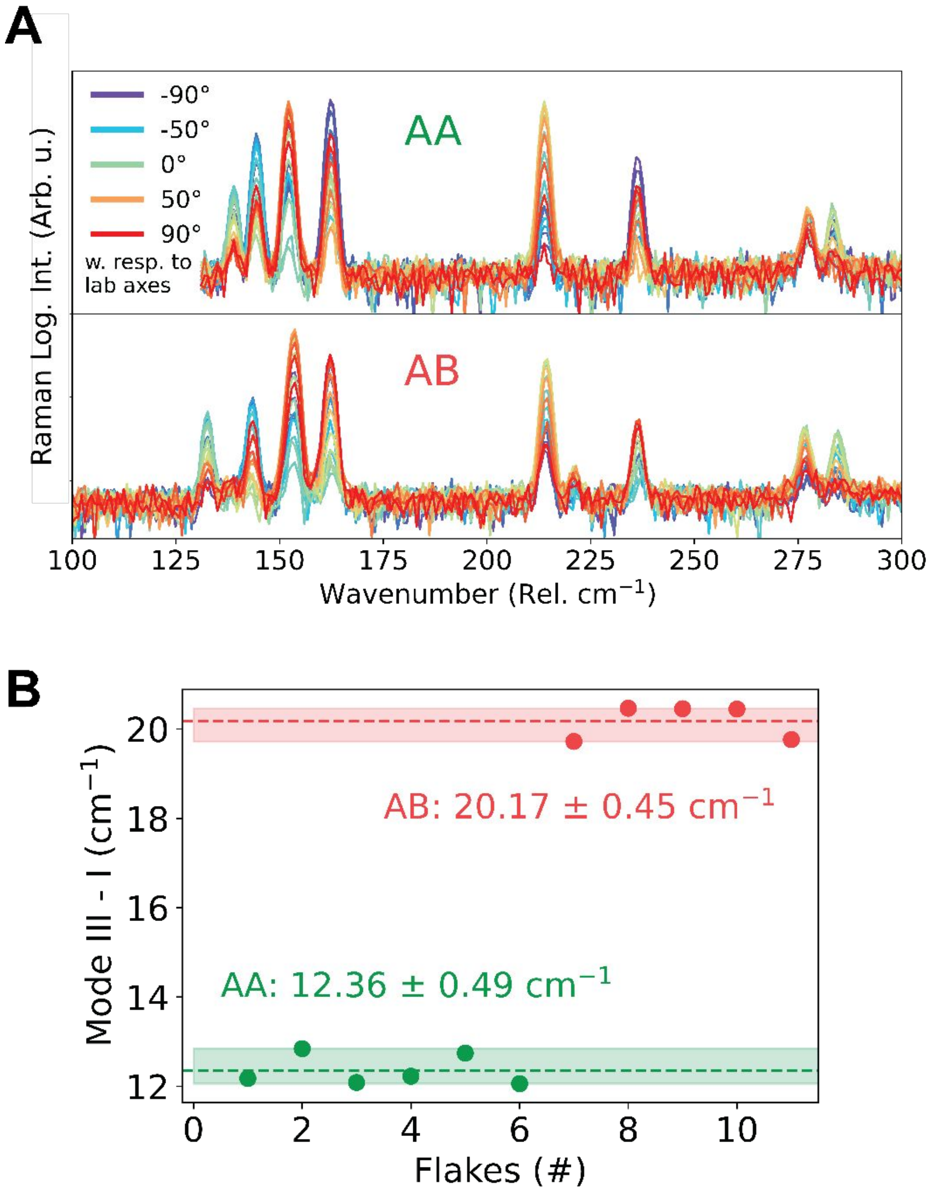

Figure S2. Full Raman spectra and flake comparisons
A) Full set of low-temperature Raman spectra of the AA and AB flakes discussed in the main text. Raman intensities are displayed on a logarithmic scale to enhance lower-intensity modes. **B)** Room-temperature Raman measurements on alternative AA and AB flakes (different from those discussed in the main text). The difference in wavenumber between modes III and I is conserved. Hence, using both different flakes, and different light polarizations (figure 1D main text) we can clearly establish whether the flake belongs to the AA- or the AB-stacking order.

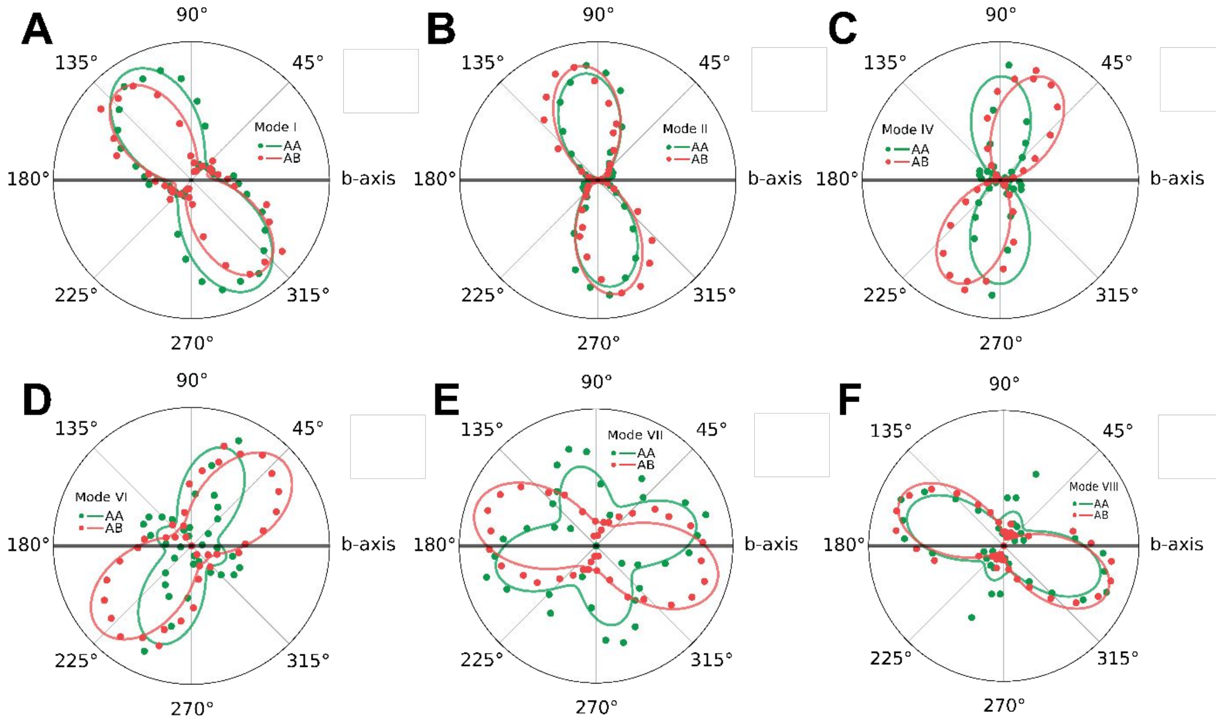

Figure S3. Raman Spectra - Supplementary polar plots
Polar plots of Raman modes not shown in the main text: Mode I **(A)**, II **(B)**, IV **(C)**, VI **(D)**, VII **(E)**, VIII **(F)**.

We note that the actual Raman tensor is more complicated than the simplified fitting with equation (1) of the main text, and the resulting fits illustrated in Fig. S3, even introducing a phase-shift, allowing for thickness-dependent behaviour. Yet, we have resorted to the simpler form here, as we found it to be sufficient for the fitting. The 4-th power in the fitting function needed to be included as the polarization behaviour of all peaks clearly shows quadrupolar behavior. While this 4th power clearly deviates from the 2nd power of the well-known Malus Law, it is consistent with the full Raman tensor description outlined in other works. [1-5]

3. Supplementary photoluminescence figures

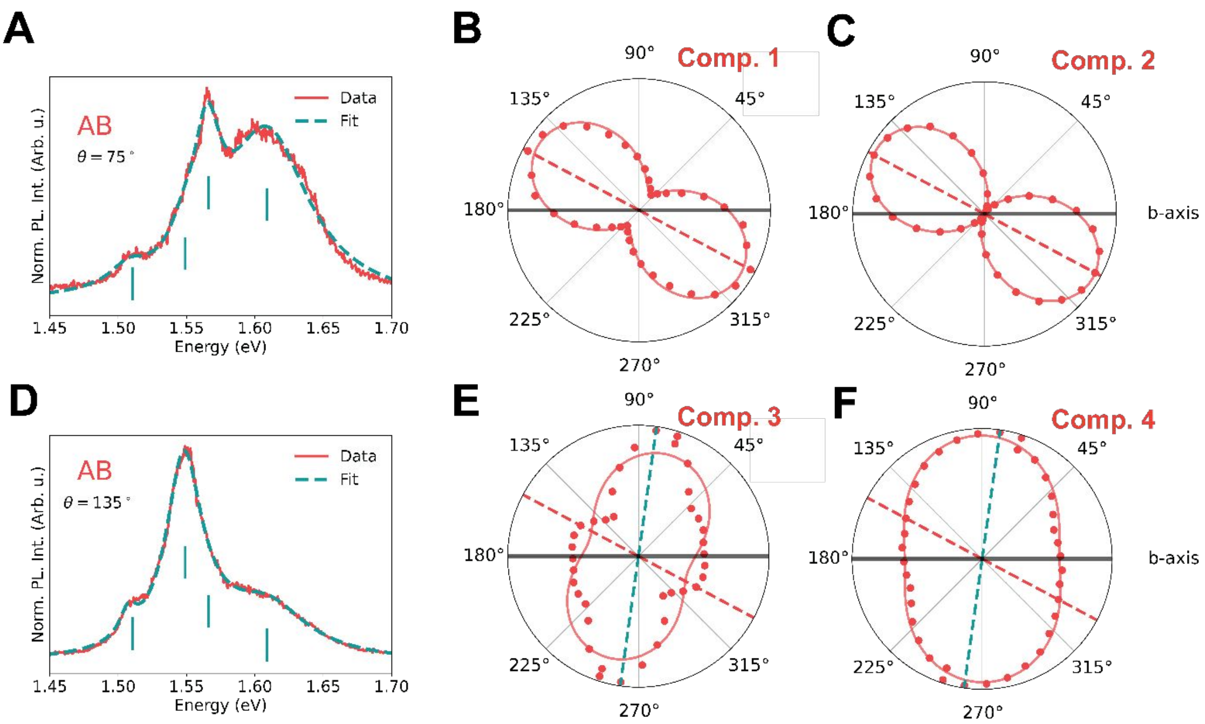

Figure S4. Four photoluminescence – spectral components and fits of AB flake (**A**,**D**) Photoluminescence spectra of AB-stacked ReS_2 measured under linear polarization angles of 75 and 135 degrees (red), and overlaid 4-component Lorentzian fit (cyan), with ticks indicating the center energies of the Lorentzians. These energies are, for all angles, 1.508, 1.548, 1.565 and 1.609 eV. The areas under the Lorentzians for the various linear polarization angles are shown on the right (**B**,**C**,**E**,**F**), with components 1 to 4 representing the Lorentzians from lower to higher energy. While the first two components (**B**,**C**) show clear exciton 1 character, on the higher energy side, the components show mixing of polarization states. In this regard, especially the third component (**E**) clearly shows mixing of exciton 1 and exciton 2 character, which prompted a deeper investigation into the polarization-dependent photoluminescence of both flakes.

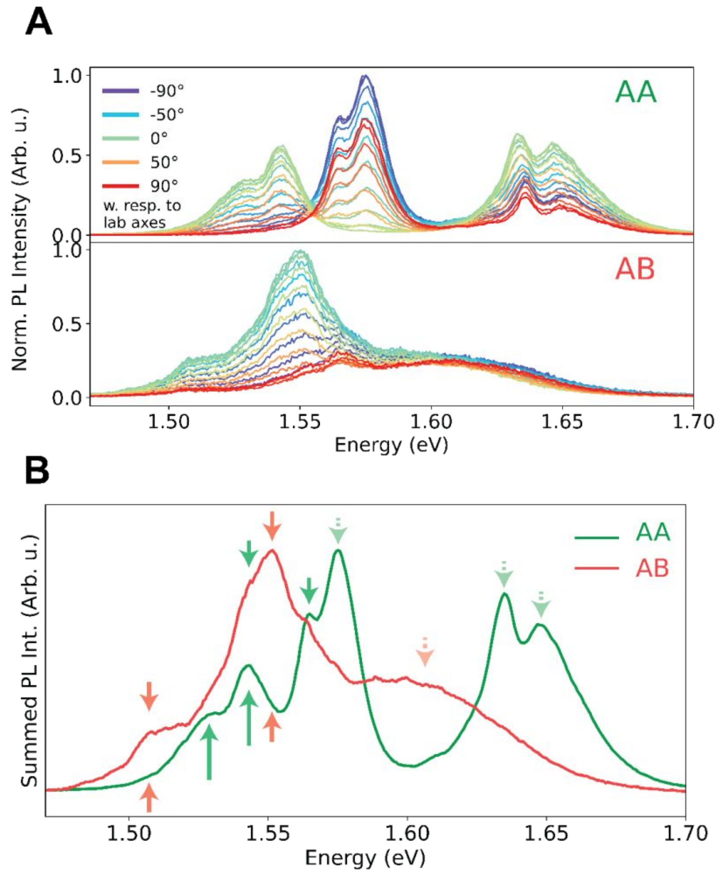

Figure S5. Linear-polarization dependent photoluminescence - supplementary data

(A) Full photoluminescence datasets for the AA (top) and AB stacking (bottom). All 19 measured linear polarization angles are plotted: -90°, -85°, and equidistant with 5° increments until 90°. Legend only labels a subset for clarity. **(B)** Photoluminescence spectra of AA and AB flakes, summed over all polarization directions. Here, the 19 datasets from panels A were background corrected, summed and normalized to effectively provide a comparison of the unpolarized photoluminescence spectra of the two stacking orders. The solid arrows indicate the spectral components considered in Fig. 3 and Fig. 4 in the main text, and the dashed/transparent arrows indicate the other spectral components that are part of the fit, for a total of 9 components. The summated plots shown here clearly reveal the presence of all peaks and shoulders in both stacking orders.

Figure S5B reveals multiple peaks and shoulders, and, in this work, we have decided to fit the full spectra with 9 components. Starting from the peaks most clear in AA stacking (green curve), the duo of peaks around 1.54 eV belong to the first linearly polarized exciton groundstate and a lower lying trion.[6] Shoulders are clearly visible in the AB spectrum (red curve) as well. The duo of peaks around 1.57 eV then correspond to the second linearly polarized exciton groundstate and its lower lying trion.[6] The peaks around 1.65 eV have been established before as higher excited states of the aforementioned ground state excitons.[7] Conversely, in the AB spectrum, the broad feature around 1.60 eV is likely composed of several peaks, but we mainly attribute it to redshifted (compared to AA) higher excited exciton states of the same groundstates mentioned above. The peak around 1.55 eV does not have a fully determined origin yet, but we argue inspired by the finding of Dhara et al. that it corresponds to a state which is optically dark in AA-stacked ReS2.[8] With our DFT calculations, detailed below, we argue that the state becomes optically bright in AB-stacked ReS2. This leaves the curious shoulder at 1.51 eV, for which we have no explanation.

4. Density Function Theory simulations

4.1 Interlayer bonding energies and bonding ranges

To evaluate the interlayer bonding energies and bonding ranges of ReS_2 flakes, we performed geometry optimization of the ReS_2 bilayers and computed their total energies employing 10 various correlation-exchange functionals, including the LDA, GGA, PBE for solid, five non-empirical vdW functionals (revPBE, DF2, optPBE, optB88 and optB86b),[9-11] and two vdW dispersion corrections (D2 and D3).[12,13] The integration in the Brillouin zone was performed using the Monkhorst-Pack scheme[14] (11×11×1) with an energy cut-off of 500 eV. The convergence threshold for the self-consistent field calculations was set to 10^{-5} eV per cell, and the geometrical structures were optimized until the Hellmann-Feynman forces acting on atoms were less than 0.01 eV/Å. The arrangement of atoms in the bilayer systems are periodic along the a and b directions. To avoid the atom interactions between the imaged periodic systems, a vacuum of 20 Å thickness was employed between the neighboring bilayers (in the c direction).

Figure S6 illustrates the optimized structures of AA- and AB-stacked ReS_2 flakes obtained with a typical vdW correction function optB86b. As shown in Table S1, the calculated lattice parameters and angles for the two ReS_2 stackings are close to each other so there should be a more sensitive parameter to resolve the optical response difference between these two materials. We consider a bilayer system containing two flat A and B layers. The total energy of this system is determined as $E = E_A + E_B + E_{interact}$, where E_A and E_B are the total energies of isolated A and B layers, respectively. $E_{interact}$ is the bonding energy of A and B. When varying the interlayer distance between A and B layers, the change of the total energy E is associated with the change of $E_{interact}$. The minimum value of $E_{interact}$ in this energy profile is considered as the interlayer bonding energy and the distance associated with this minimum is considered as the interlayer distance of this bilayer material. Upon further separating the layers, $E_{interact}$ grows.

We now expand the model above to the ReS_2 bilayer flakes. Since each ReS_2 layer is not flat, to obtain the interlayer bonding energy and bonding range of ReS_2 bilayers, we first define the interlayer distance *d* as the distance between two layers along the direction of the lattice vector c (Fig. S7a). The purple plane contains the highest atom of the lower layer while the green plane contains the lowest atom of the upper layer. The perpendicular interlayer distance $d\perp$ is defined as the distance of these two planes. To obtain the interaction energy profile as shown in Fig. S7b for a typical optB86b exchange-correlation functional, we move the upper layer along the c-direction while fixing the lower layer, and calculate $E_{interact}$ for each single variation. As shown in Fig. S7c, E_b increases when Van der Waals corrections are considered, suggesting that these materials are sensitive to the long-range Van der Waals attraction. The resulting interlayer distances d are around 2.6-2.8 Å in AA- and AB-stacked ReS_2 (Fig. S7d).

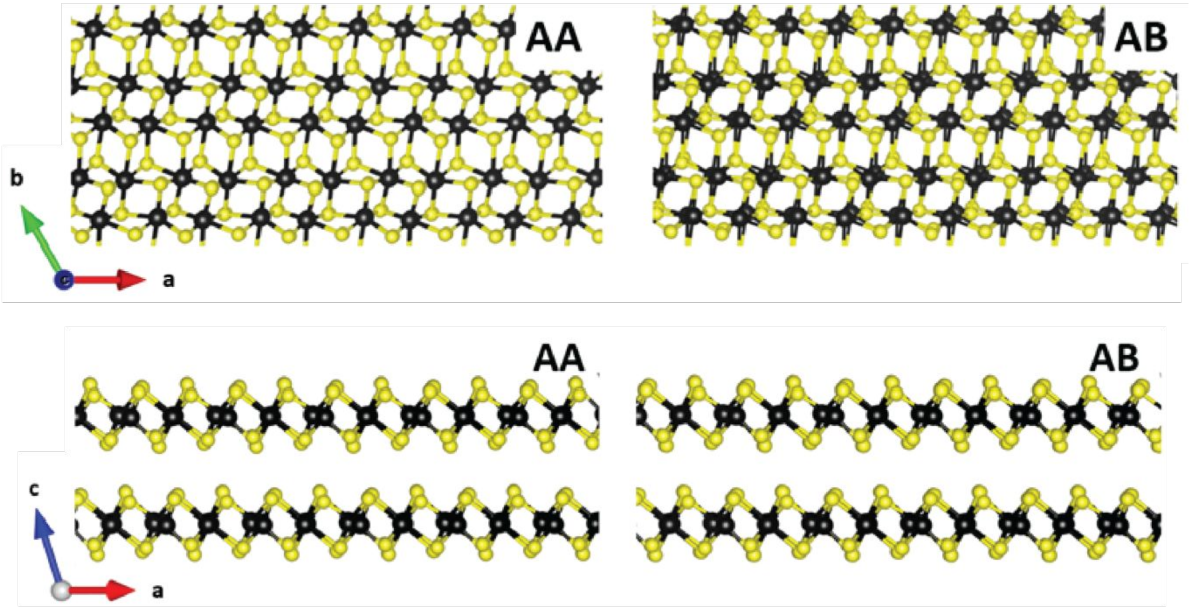

Figure S6. Top view and side view of AA and AB stacking of ReS_2 bilayers. Re atoms are in black and Se atoms are in yellow. The crystal axes are indicated on the left of the figures. The side view of AB shows that the 2 layers are displaced by half of a unit cell along the a-axis. These parameters are obtained by optB86b functional.

Table S1. Calculated lattice parameters and angles for ReS_2 bilayer systems. These parameters are obtained by optB86b functional.

	a (Å)	b (Å)	d (Å)	d⊥ (Å)	α (°)	β (°)	γ (°)
AA stacking	6.381	6.482	2.612	2.610	91.736	104.062	118.838
AB stacking	6.381	6.482	2.631	2.627	92.491	103.886	118.841

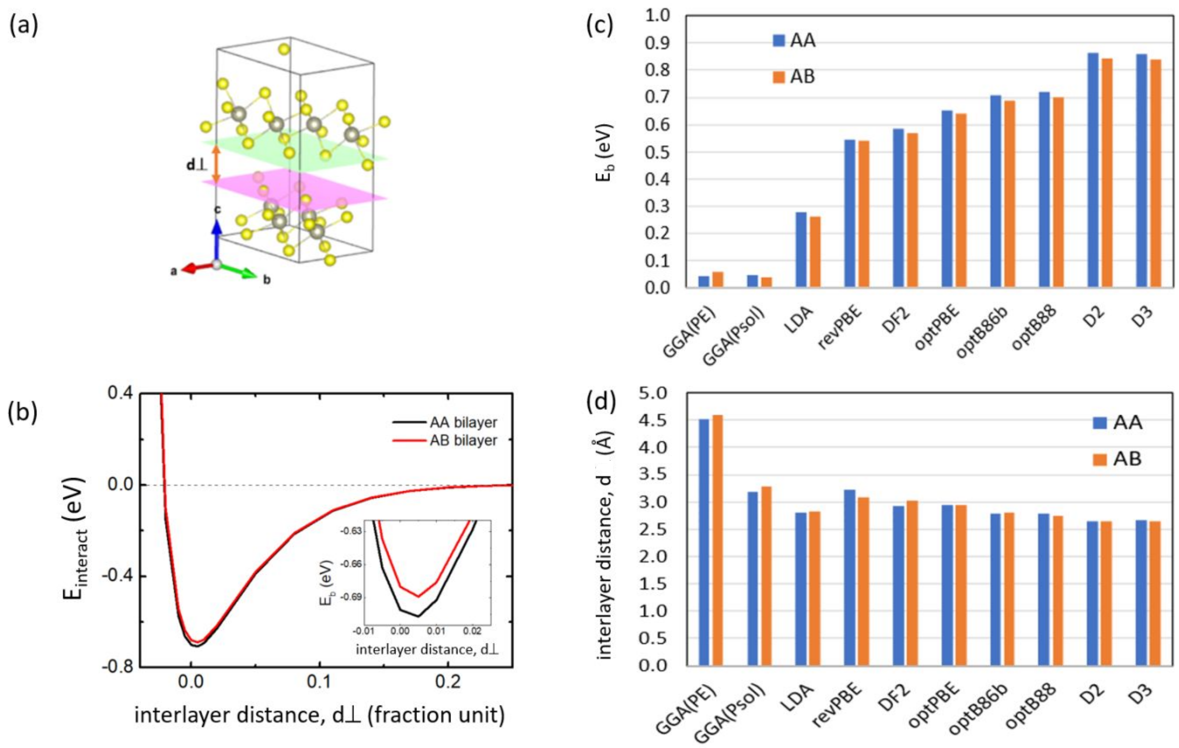

Figure S7. Interlayer bonding energies and interlayer distances of ReS_2 bilayers. (a) A sketch of interlayer distance definition. (b) A typical interlayer energy profile for optB86b exchange-correlation functional. Calculated interlayer bonding energies (c) and interlayer distances 'd' (d) for AA and AB stacking orders using various functionals. In general AA and AB show similar bonding strengths and interlayer distances as can be observed by the agreement in the minima.

4.2 Potential energy landscape

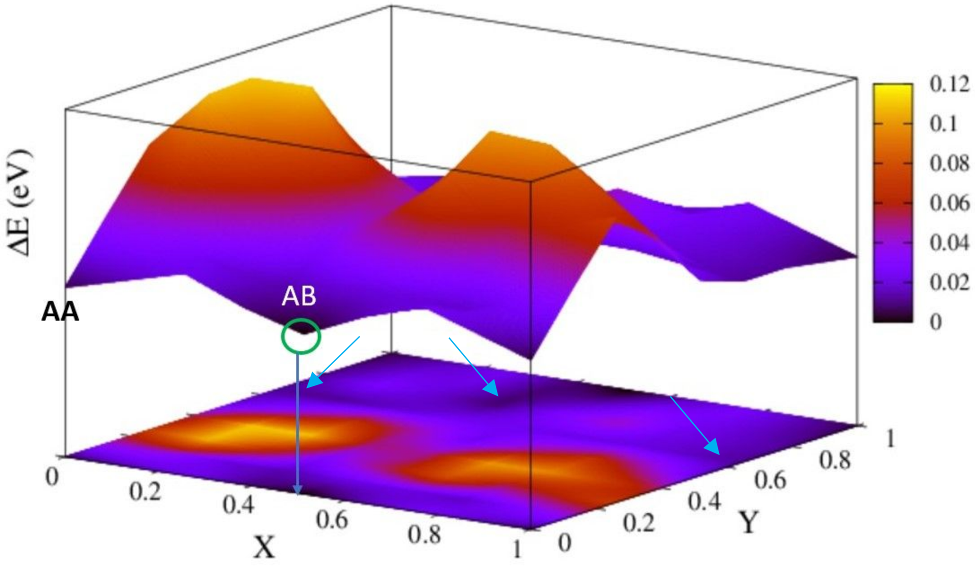

Figure S8. Interlayer interaction energy on the (110) plane of a conventional unit cell of ReS_2. The upper is the 3D-potential energy surface and the lower is its projected image on the (110) plane. The interaction energy values are color-coded with a color map on the right-hand side. AB-stacked structure is found at the fractional coordinate of (0.5,0). These parameters are obtained by optB86b functional.

Fig. S8 shows the three-dimensional potential energy surface and its two-dimensional projection obtained using optB86b van der Waals functional. The X- and Y-axes represent the fractional coordinates along the *a* and *b* lattice vectors of the ReS_2 unit cell. The AA stacking configurations are located at (0,0) while the AB configurations are at (0.5, 0). The color map represents the relative values of the potential energy surface (PES): dark color regions of the XY-projected PES indicate the most favorable stacking configurations. Besides the AA and AB well-accepted ReS_2 stacking sequences, our method also suggests other potential configurations, whose energies are only several tens of meV higher than the AA stacking as indicated by arrows in Fig. S8.

4.3 Spin-orbit coupling band structures of AA and AB flakes with optB86b functionals

Fig. 5 in the main text shows the band structures of AA and AB ReS_2 bilayers calculated by the optB86b-vdW functional including the spin-orbit coupling effect. Both AA- and AB-bilayers exhibit direct band gaps at the Gamma point with band gap values of ~1.45 eV and ~1.35 eV, respectively. Note that, without the spin-orbit coupling effect, the band gap of bilayers is ~1.45 eV for both cases. Interestingly, the two highest occupied levels at the Gamma point change the spin characteristics when the AA-bilayer is deformed to AB-bilayer. Specifically, in the band structure of AA-bilayer stacking, the highest singly occupied molecular orbital (SOMO) level is occupied by an electron with spin down while the next SOMO level is occupied by an electron with spin up. In contrast, for AB-bilayer stacking, the occupied spins are reversed for the two highest SOMO levels. The spin reversal only happens at the valence band maxima while at the conduction band minima the spin directions of both stackings remain unchanged. Furthermore, as shown in Fig. S10, this interesting spin flips are mainly caused by the *d*-orbital of the Re atoms close to the Fermi level. By analogy, in other transition metal dichalcogenide materials, such as WS_2 and WSe_2, the spin-split band indeed correspond to exciton energy splitting in bulk and single layers.[13,14] In these other TMDC systems the precise energy of spin projected bands are also dependent on the exchange interaction, more work is necessary in the case of ReS_2 to determine whether the spin splitting results in lower lying dark states or not, explaining the differences in AA and AB in more detail.

4.4 Effective mass determination from the bandstructures

We use the calculated electronic bandstructures shown in Fig. 5 of the main text to extract the band dispersions around specific k-points of the Brillouin zone. Specifically, the dispersions along the $M-\Gamma-M$ and $K-\Gamma-K$ paths, corresponding to the k_x and k_y directions mentioned in table S2, were extracted by fitting the bands with a second order polynomial. Through the fitting function we obtain the coefficient of the second order term, $A_0 = (\partial^2 E/\partial k^2)$, and obtain the effective masses as,

$$m_{e,h}^* = \frac{\hbar^2}{2 * A_0} \qquad \text{(S3)}$$

where $m_{e,h}^*$ are the effective masses of the electrons and holes in their respective bands and $\hbar$ is the reduced Planck constant. The reduced excitonic masses are then obtained with,

$$\mu = \frac{m_e^* m_h^*}{m_e^* + m_h^*} \qquad \text{(S4)}$$

where the reduced exciton mass depends on the chosen spin-split hole band. For AA reduced exciton masses it is the lower, flatter, band that has effective hole mass m_{h2}^* and for AB the exciton mass was determined from the effective electron mass, m_e^*, and the effective hole mass, m_{h1}^*.

As can be seen from table S2, the reduced exciton masses of excitons dominant in the AB stacking are smaller than those of AA ReS_2, in both M and K directions. This translates into a smaller exciton binding energy for these excitons, consistent with our experimental results. It is worth noting that the reduced exciton masses have been estimated before, from experimental data, for the AA stacking and the values found there are consistent with ours.[7]

	AA		AB	
	x	*y*	*x*	*y*
m_e^*	0.430	0.510	0.419	0.479
m_{h1}^*	1.901	1.594	2.077	1.724
m_{h2}^*	2.409	2.002	2.467	2.177
μ	0.365	0.406	0.348	0.375

Table S2. Determined effective masses
The elements of the table give the effective masses of the quasi-electron (m_e^*) and the quasi-holes (m_{h1}^*, m_{h2}^*) in units of the free-electron mass (m_e), for the x,y directions in reciprocal space in the cases of AA- and AB-stacked ReS_2. It is clear that the second hole effective mass is bigger than the first in all cases. The reduced masses (μ) are calculated from m_{h2}^*, m_e^* for the optically allowed transition in the AA variant, and m_{h1}^*, m_e^* in the case of AB stacked ReS_2. These calculations show that along both directions, AB has a lower reduced exciton mass than AA, translating into lower exciton binding energies.

4.5 Partial density of states

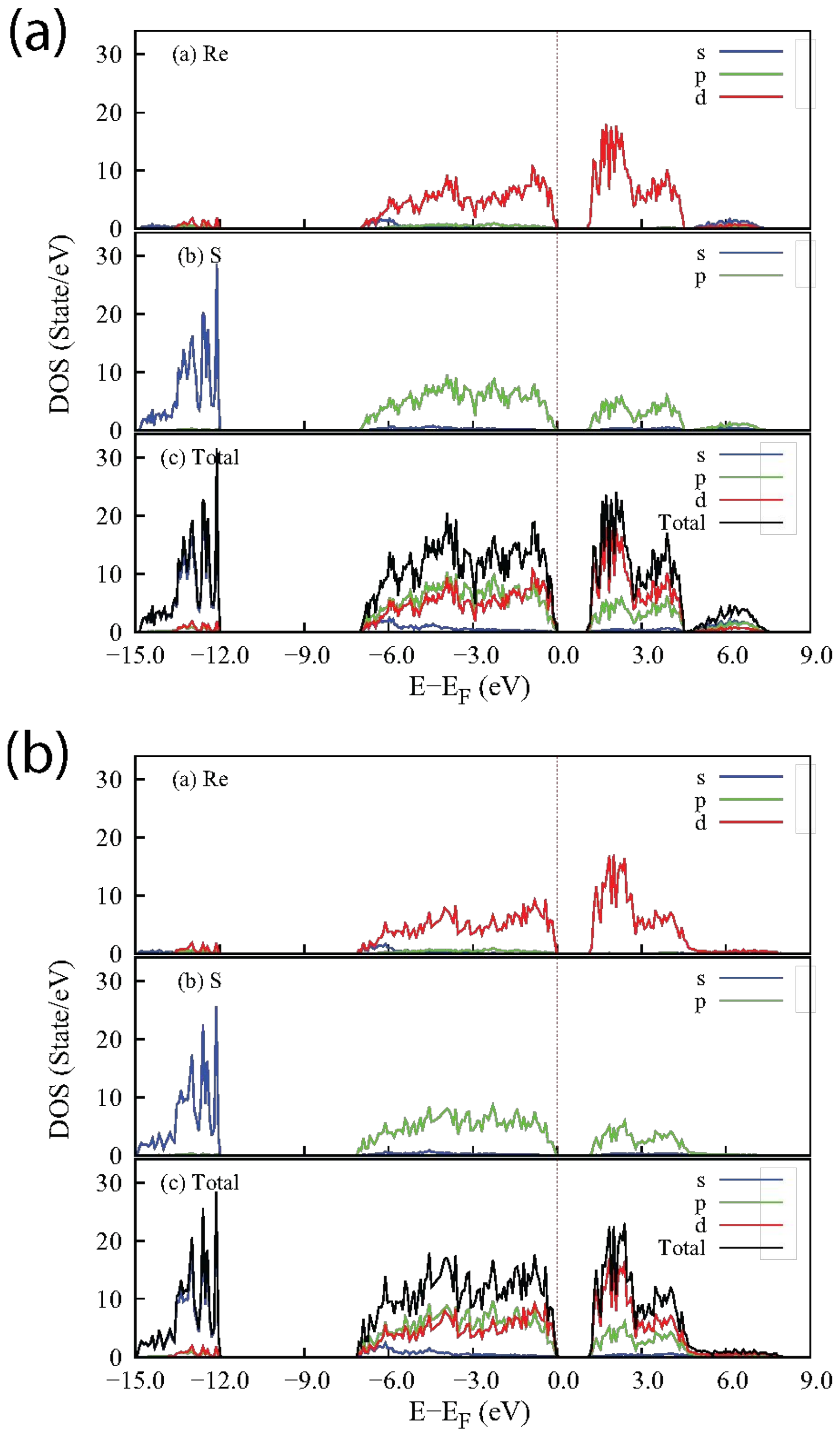

Figure S9. Projected density of states of AA (a) and AB (b) ReS_2 bilayers. The colors illustrate the specific orbital contribution to the total projected density of states. These calculations are obtained by optB86b functional.

5. References

(1) Kranert, C., Sturm, C., Schmidt-Grund, R. and Grundmann, M. Raman Tensor Formalism for Optically Anisotropic Crystals. *Phys. Rev. Lett.* **2016**, 116(12), p.127401.
(2) Zhang, S., Mao, N., Zhang, N., Wu, J., Tong, L. and Zhang, J. Anomalous Polarized Raman Scattering and Large Circular Intensity Differential in Layered Triclinic ReS2. *ACS Nano* **2017**, 11(10), pp.10366-10372
(3)Hart, L., Dale, S., Hoye, S., Webb, J.L. and Wolverson, D. Rhenium Dichalcogenides: Layered Semiconductors with Two Vertical Orientations. *Nano Lett.* **2016**, 16(2), pp.1381-1386.
(4) McCreary, A., Simpson, J.R., Wang, Y., Rhodes, D., Fujisawa, K., Balicas, L., Dubey, M., Crespi, V.H., Terrones, M. and Hight Walker, A.R. Intricate Resonant Raman Response in Anisotropic ReS2. *Nano Lett.* **2017**, 17(10), pp.5897-5907.
17(5), pp.3202-3207.
(5) Choi, Y., Kim, K., Lim, S. Y., Kim, J., Park, J. M., Kim, J. H., ... & Cheong, H. Complete determination of the crystallographic orientation of ReX 2 (X= S, Se) by polarized Raman spectroscopy. *Nanoscale Horiz.* **2020**, 5(2), 308-315.
(6) Wang, X., Shinokita, K., Miyauchi, Y., Cuong, N.T., Okada, S. and Matsuda, K. Experimental Evidence of Anisotropic and Stable Charged Excitons (Trions) in Atomically Thin 2D ReS2. *Adv. Funct. Mater.* **2019**, 29(51), p.1905961.
(7) Jadczak, J., Kutrowska-Girzycka, J., Smoleński, T., Kossacki, P., Huang, Y.S. and Bryja, L. Exciton Binding Energy and Hydrogenic Rydberg Series in Layered ReS2. *Sci. Rep.* **2019**, 9(1), pp.1-9
(8) Dhara, A., Chakrabarty, D., Das, P., Pattanayak, A. K., Paul, S., Mukherjee, S., & Dhara, S. Additional excitonic features and momentum-dark states in ReS 2. *Phys. Rev. B* **2020** 102(16), 161404.
(9) Klimeš, J., Bowler, D.R. and Michaelides, A. Van Der Waals Density Functionals Applied to Solids. *Phys. Rev. B* **2011**, 83(19), p.195131.
(10) Klimeš, J., Bowler, D.R. and Michaelides, A. Chemical accuracy for the van der Waals density functional. *J. Phys. Condens. Matter* **2009**, 22(2), p.022201.
(11) Lee, K., Murray, É.D., Kong, L., Lundqvist, B.I. and Langreth, D.C. Higher-Accuracy Van Der Waals Density Functional. *Phys. Rev. B* **2010**, 82(8), p.081101.
(12) Bucko, T., Hafner, J., Lebegue, S. and Angyan, J.G. Improved Description of the Structure of Molecular and Layered Crystals: Ab Initio DFT Calculations with Van Der Waals Corrections. *J. Phys. Chem. A* **2010**, 114(43), pp.11814-11824.
(13) Grimme, S., Antony, J., Ehrlich, S. and Krieg, H. A Consistent and Accurate Ab Initio Parametrization of Density Functional Dispersion Correction (DFT-D) for the 94 Elements H-Pu. *J. Chem. Phys.* **2010**, 132(15), p.154104.
(14) H. J. Monkhorst and J. D. Pack, Special Points for Brillouin-Zone Integrations, *Phys. Rev. B* **1976**, 13(12), p. 5188–5192
(15) Tran, Q. H.; Tran, T. N.; Luong, T.; Ngo, V. T.; Phung, V. B.; Dinh, V. A. Band Valley Flattening and Exciton Appearance/Disappearance under Isotropic Strain in Monolayer WS2. *Eur. Phys. J. Plus* **2022**, 102, 1317.
(16) Tran, T.N., Dang, M.T., Tran, Q.H., Luong, T.T. and Dinh, V.A. Band Valley Modification under Strain in Monolayer WSe2. *AIP Adv.* **2022**, 12(11), p.115023.
